# Microbiota and Cancer: The Emerging Beneficial Role of Bifidobacteria in Cancer Immunotherapy

**DOI:** 10.3389/fmicb.2020.575072

**Published:** 2020-09-08

**Authors:** Giulia Longhi, Douwe van Sinderen, Marco Ventura, Francesca Turroni

**Affiliations:** ^1^Laboratory of Probiogenomics, Department of Chemistry, Life Sciences, and Environmental Sustainability, University of Parma, Parma, Italy; ^2^Alimentary Pharmabotic Centre (APC) Microbiome Institute and School of Microbiology, Bioscience Institute, National University of Ireland, Cork, Ireland; ^3^Microbiome Research Hub, University of Parma, Parma, Italy

**Keywords:** microbiota, cancer, *Bifidobacterium*, microbial biomarker, probiotics

## Abstract

Many intestinal bacteria are believed to be involved in various inflammatory and immune processes that influence tumor etiology because of their metabolic properties and their ability to alter the microbiota homeostasis. Although many functions of the microbiota are still unclear, there is compelling experimental evidence showing that the intestinal microbiota is able to modulate carcinogenesis and the response to anticancer therapies, both in the intestinal tract and other body sites. Among the wide variety of gut-colonizing microorganisms, various species belonging to the *Bifidobacterium* genus are believed to elicit beneficial effects on human physiology and on the host-immune system. Recent findings, based on preclinical mouse models and on human clinical trials, have demonstrated the impact of gut commensals including bifidobacteria on the efficacy of tumor-targeting immunotherapy. Although the underlying molecular mechanisms remain obscure, bifidobacteria and other microorganisms have become a promising aid to immunotherapeutic procedures that are currently applied to treat cancer. The present review focuses on strategies to recruit the microbiome in order to enhance anticancer responses and develop therapies aimed at fighting the onset and progression of malignancies.

## General Features of the Gut Microbiota

The definition of microbiome and microbiota is rather complex and often these two terms are used interchangeably. The microbiota represents the entire population of microorganisms colonizing a specific ecological niche, whereas the microbiome encompasses the full genetic complement of an entire microbiota (Ursell et al., 2012b). In recent years, many studies have focused on the analysis of the bacterial composition that inhabits various sites of the human body. In particular, the Human Microbiome Project (HMP), based on the concept that we are organisms made up of a large number of human and bacterial cells, aims to define the microbiome that consists and/or influences our metabolism, our physiology and any predispositions to diseases (Turnbaugh et al., 2007). The currently employed molecular techniques applied to the microbiota analysis, including the recently emerged metagenomic technology, are based on culture-independent methods. Their application have been made possible due to the advancement of next-generation sequencing methods (NGS), allowing the compositional evaluation of bacterial populations and the discovery of essentially the entire genetic blueprint of microbial communities (i.e., microbiota and microbiome analysis) (Mancabelli et al., 2020).

The human microbiota comprises trillions of symbiotic microbial cells, present in different areas of the body. The majority of these are located in the intestine where they are involved in various functions including nutrient assimilation, vitamin synthesis, bile acid/salt and sterol metabolism, immune stimulation, and maintenance of intestinal homeostasis. Given the variety and importance of such functions, the intestinal microbiota operates as a separate organ of the human and animal superorganism (Brestoff and Artis, 2013; Guinane and Cotter, 2013; Molinero et al., 2019; Illiano et al., 2020).

The differences in bacterial composition in each microbial habitat are due to different environmental conditions such as pH, oxygen levels/redox state, availability of nutrients, humidity and temperature. All these environmental features allow various populations to thrive and exert different activities, while interacting with the (human) host environment (Ursell et al., 2012b).

The composition of the human intestinal microbiota is very complex and includes bacteria, archaea, fungi and viruses that have adapted to live on the mucous surface of the intestine or in its lumen (Nuriel-Ohayon et al., 2016), developing immediately after birth and varying between different gut locations, between individuals and over time. Until today, it has been assumed that the neonatal gut intestine was a sterile niche up until birth (Putignani et al., 2014), though various scientific reports have questioned this notion, claiming that bacteria are present in the gut before birth (Nuriel-Ohayon et al., 2016). However, a growing number of scientific publications have argued against such a possibility and most evidence currently favors the idea of a sterile placenta (Lauder et al., 2016). The period immediately following birth is deemed to be crucial for the appropriate development of the gut microbiota (Turroni et al., 2020). Vaginal delivery and breastfeeding are the main defining factors that favor efficient and correct microbial colonization events of the neonatal gastrointestinal tract (Milani et al., 2017a). Among the first colonizers of the infant gut microbiota are bifidobacteria (Turroni et al., 2012), rapidly populating the infant gut within the first weeks following birth. This remarkable phenomenon of gut colonization is believed to be at least partially dependent on the bifidogenic activities of specific mother milk-derived oligosaccharides, commonly referred to as human milk oligosaccharides (HMOs) (Turroni et al., 2019). Recent studies have shown that the bifidobacteria present in the mother’s gut microbiota strongly correlates with that of her baby, indicative of vertical transmission of bacteria from mother to baby (Rautava et al., 2012; Nuriel-Ohayon et al., 2016).

The transition to complementary feeding, and therefore the introduction of solid foods, favors the differentiation of the intestinal microbiota and increases microorganisms belonging to the families of *Lachnospiraceae*, *Ruminococcaceae*, *Eubacteriaceae*, *Rikenellaceae*, and *Sutterellaceae* (Laursen et al., 2016). During subsequent years, the microbiota develops to form its adult state and tends to maintain this homeostasis (Underhill and Iliev, 2014), which means that the microbiota composition of a healthy adult gut is stable (Rodriguez et al., 2015). The intestinal bacterial profile in adulthood displays a high level of inter-individual variability, being influenced by a wide range of factors such as health status, dietary habits, use of antibiotics or other drugs, age, genetics, ethnicity and geography (Ursell et al., 2012b; Yatsunenko et al., 2012). The main bacterial phyla of the human gut microbiota encompass members of the *Firmicutes*, *Bacteroidetes*, *Actinobacteria*, *Proteobacteria*, *Tenericutes*, and *Fusobacteria*. Notably, the gut microbiota of adults are dominated by Firmicutes and Bacteroidetes, which together make up 90% of the human gut microbiota (Rajilic-Stojanovic et al., 2007). The adult gut microbiota composition is radically different from that of the infant’s intestine, in which Actinobacteria, and in particular bifidobacteria, are commonly the most numerous microorganisms (Turroni et al., 2012). In addition, the adult microbiota has proven to be more complex than that of infants in terms of the total number of bacteria and microbial diversity (Eckburg et al., 2005). The microbiota composition changes with aging and becomes less complex in terms of number of species and relative abundance in the elderly population (Claesson et al., 2011). Throughout life, diet influences bacterial colonization and persistence in the intestine, thus shaping the gut microbiota composition (Fuentes and de Vos, 2016). In this context, butyrogenic bacteria such as members of the genus *Clostridium* cluster XIVa, responsible of butyrate production, are more abundant in the fecal microbiota of omnivores than in the vegetarian microbiota, including humans. However, in response to a Western-based diet, which is characterized by the presence of low fiber levels and high fat intake, the bacteria responsible for fiber degradation, such as *Prevotella*, *Succinivibrio*, *Treponema*, and *Bifidobacterium*, are reduced in abundance. Conversely, a diet mainly based on meat causes an increase of bile-tolerant bacteria (e.g., *Alistipes*, *Bilophila*) to the detriment of the microorganisms involved in the metabolism of plant polysaccharides (*Firmicutes*) (Milani et al., 2016).

Gut microbiota plays a key role in maintaining and supporting human health. Any deviation from its “normal” composition, a condition for which the generic term dysbiosis was coined (Tamboli et al., 2004), is believed to herald the onset or the worsening of certain diseases, including autoimmunity, colorectal cancer, metabolic diseases, and bacterial infections (Prakash et al., 2011). Indeed, recent work has indicated that altered microbial communities and intestinal barrier impairment are associated with the development of a number of chronic inflammatory disorders, including inflammatory bowel disease (IBD), celiac disease, multiple sclerosis, rheumatoid arthritis, psoriasis, type 2 diabetes, allergic diseases, cardiovascular, and neurodegenerative diseases (Yu, 2018), some of which may directly or indirectly lead to cancer (Stidham and Higgins, 2018).

## The Role of Microbiota in Carcinogenesis

Through their metabolic activities, intestinal bacteria are believed to influence various inflammatory and immune processes that are implicated in tumor etiology, such as in colorectal cancer (CRC) (Kinross et al., 2011; Clemente et al., 2012). CRC is one of the major causes of mortality in developing countries (Jemal et al., 2011). Even though it is well-established that a healthy environment and lifestyle reduce the risk of carcinogenesis, it is still extremely difficult to identify the triggering factor(s) of cancer in individuals, due to its multifactorial etiology (Hanahan and Weinberg, 2011). Currently, the incidence of cancer is still increasing, possibly and in part due to cancer-associated lifestyle choices such as smoking, “westernized” diet and physical inactivity. However, increased exposure to known carcinogens or suspected carcinogens may also be a contributing factor (Torre et al., 2015). Cancer may therefore result from the impact of various genetic factors acting in concert with a range of environmental and life-style associated insults (Garrett, 2015). Studies involving germ-free animals, i.e., animals without a resident intestinal microbiota, have provided compelling evidence for tumor-promoting effects of the microbial composition in spontaneous, as well as genetically or carcinogen-induced tumorigenesis in various organs (Schwabe and Jobin, 2013). Germ-free mice exhibit severe defects in their immunity system, with a near-absent mucous layer and altered IgA secretion (Gopalakrishnan et al., 2018a). Similarly, depletion of the intestinal bacterial microbiota in mice by means of antibiotic treatment, reduces the development of cancer in the liver and in the colon (Dapito et al., 2012; Yoshimoto et al., 2013). It has been suggested that common microbial inhabitants of the human gut, such as *Escherichia coli*, which normally coexist harmoniously with their mammalian host and promote intestinal homeostasis, may sometimes facilitate colorectal carcinogenesis (Cuevas-Ramos et al., 2010). Indeed, some virulent *E. coli* strains with acquired pathogenicity islands encoding for a multi-enzymatic machinery for the production of a peptide-polyketide hybrid genotoxin named colibactin, can colonize the human gastrointestinal tract and cause gut diseases (Sun and Kato, 2016). These particular *E. coli* strains are more commonly present in the mucosa of CRC and IBD patients and they induce double-strand DNA breaks, mutations and chromosomal rearrangements. They also modulate the tumor microenvironment favoring the emergence of senescent cells, which may affect tumor promotion and cancer progression via the secretion of growth factors (Dalmasso et al., 2014).

In addition, a recent report has demonstrated that intestinal bacteria belonging to the class of Gammaproteobacteria can influence the efficacy of cancer therapies by metabolizing the chemotherapeutic drug gemcitabine into its inactive form, commonly used to treat pancreatic ductal adenocarcinoma (PDAC) (Geller et al., 2017). Moreover, thanks to the current knowledge on the role of gut microbes in gastrointestinal carcinoma development, novel approaches targeting the gut microbiota represent a promising way to prevent cancer or at least to delay cancer cell proliferation (Brennan and Garrett, 2016). Therefore, the gastrointestinal microbiota appears to play opposing roles in both preventing and promoting carcinogenesis.

One of the main activities of the colonic intestinal microbiota is to acquire energy by fermenting dietary elements (e.g., polysaccharides) that are not metabolized by host enzymes or by the microorganisms residing in the upper gastrointestinal tract (GIT) (Rowland et al., 2018). Many of such indigestible carbohydrates, resistant to human digestion, enter the colon where they are metabolized by resident microbiota into short chain fatty acids (SCFAs) such as butyrate, propionate and acetate, which are in turn absorbed by the intestinal epithelial cells (IECs) through passive diffusion (Pryde et al., 2002). SCFAs, and in particular butyrate, represent the primary energy source for IECs and play an important role in maintaining the integrity of the associated epithelial layer (Lauder et al., 2016).

Furthermore, butyrate is a plausible candidate for tumor suppression and prevention because it inhibits cell proliferation and induces cell differentiation or apoptosis when added to tumor-derived cell lines (Hamer et al., 2008; Fung et al., 2012). In addition, some lactic acid bacteria (LAB) have been proposed to confer benefits to the host by influencing metabolic, immunological and protective functions in the colon (Marteau et al., 2001). In animal models, treatment with certain LAB was shown to prevent carcinogen-induced pre-neoplastic lesions or tumors (Wollowski et al., 2001). Besides, it has also been demonstrated that particular LAB species are involved in the detoxification of certain carcinogens such as polycyclic aromatic hydrocarbons (PAH) and heterocyclic aromatic amines (Knasmuller et al., 2001; Hope et al., 2005). PAH may also damage DNA of colonocytes (Diggs et al., 2011). However, the mechanism by which these bacteria achieve inactivation of carcinogens remains unclear; it may be that certain gut commensals catalyze detoxification reactions and/or produce metabolites that cause carcinogen detoxification (Rafter, 2003).

Furthermore, gut microorganisms that lack the ability to produce butyric acid may influence the growth of butyrogenic microorganisms by synthetizing metabolites that are specifically utilized by these bacteria. In this respect, bifidobacteria synthesize various organic acids, such as acetic acid that can be used as a metabolic precursor for butyric acid biosynthesis by butyrogenic microorganisms such as *Faecalibacterium prausnitzii* and *Eubacterium rectale* (Waddington et al., 2010).

As mentioned above, bacteria belonging to the *Clostridium* genus, despite exerting physiologically important effects on the colonic epithelium and on the host metabolism of omnivores, are known to convert bile acids into secondary products such as deoxycholic acid (DCA), which is a known carcinogen (Knasmuller et al., 2001; Staley et al., 2017). This finding shows how intestinal microorganism sometimes play a key role in the activation and detoxification of various classes of carcinogens, thereby influencing cancer risk for individuals (Hambly et al., 1997).

Many tumor-promoting effects of the microbiota, not only in colorectal cancer but also in other cancer types, are caused by altered host-microbiota interactions and dysbiosis. The microbiological imbalance, caused by the failure of some control mechanisms like barrier defects, immune defects and loss of eubiosis, may cause a modification of intercellular tight junctions (Llopis et al., 2009), in turn causing effective penetration of antigens responsible for the activation of gut associated lymphoid tissue (GALT) with consequent tissue damage (Arseneau and Cominelli, 2009). These combined factors enhance the chances of pathogenic bacteria to encourage carcinogenesis under particular conditions. In this context, infection with *Helicobacter pylori*, which is classified as a carcinogenic microorganism by the International Agency for Research on Cancer (IARC), may lead to the sequential development of gastritis, gastric ulcer, atrophy and finally gastric cancer (Fox and Wang, 2007). However, gastric cancer is also promoted by the presence of a complex microbiota. This phenomenon was identified in murine models treated with *H. pylori*, which developed fewer tumors than their pathogen-free counterparts. This is probably due to the ability of *H. pylori* to provoke gastric atrophy and hypochlorhydria, which causes the stomach being susceptible to bacterial overgrowth, and subsequently increased bacterial conversion of dietary nitrates into carcinogens (Lofgren et al., 2011).

Besides, dysbiosis is regarded as one of the highest risk factors of chronic inflammation through immune system activation (Fujimura et al., 2010). Rudolf Virchow first suggested the connection between inflammation and cancer in 1863, when he observed the presence of leukocytes within tumor tissues and hypothesized that the presence of these cells mirrored the origin of the tumors in sites characterized by chronic inflammation (Virchow, 1989). IBDs, including Crohn’s disease (CD), are genetically linked to a phenotype characterized by a significant reduction of the microbiota complexity, an increased abundance of *Enterobacteriaceae*, *Pasteurellaceae*, *Fusobacteriaceae*, *Neisseriaceae*, *Veillonellaceae*, and *Gemellaceae*, and decreased abundance of *Bifidobacteriaceae*, *Erysipelotrichaceae*, *Clostridiales*, and *Bacteroidales* (Gevers et al., 2014). *Faecalibacterium prausnitzii*, which represents about 5% of the intestinal microbiota in healthy adults, is also a widely recognized microbial marker associated with IBD (Martin et al., 2017). Low levels of *F. prausnitzii* in fecal and mucosal samples have been shown to be predictive of both incidence and recurrence of IBD (Sokol et al., 2008, 2009). This is mainly due to the critical role played by this bacterium in maintaining intestinal homeostasis and health through regulation of the metabolic activity of colonocytes (Scheppach, 1994) and the integrity of the mucous layer (Wrzosek et al., 2013).

It is well-established that individuals with IBD have a higher risk of developing gastrointestinal cancer, with risk level corresponding to the duration and severity of mucosal inflammation (Hope et al., 2005). The increased risk of cancer in IBD patients may be associated with the chronic cellular proliferation required to repair damage to the epithelial monolayer caused by constant inflammation. In chronic inflammation, cytokines secreted by immune cells stimulate the pathways that are also connected to cancer proliferation (Morgillo et al., 2018). For example, tumor necrosis factor-alpha (TNF-α) and interleukin 6 (IL-6), the main cytokines released during chronic inflammation, are known to stimulate proliferation of cancer cells, their survival and their dissemination.

## Cancer and Novel Microbial Markers

As described above, there is accumulating scientific evidence that certain members of the microbiota are implicated in tumor development. One bacterium that has recently attracted the interest of the scientific community based on studies of the microbiome of colorectal cancer is *Fusobacterium nucleatum* (Mima et al., 2016). This microorganism belongs to the Fusobacteria phylum, which are Gram-negative, non-spore-forming, typically non-motile anaerobes with a tapered rod shape (Brennan and Garrett, 2019). Among those species colonizing humans, *F. nucleatum* is the most abundant in the oral cavity and a common member of the oral microbiota, playing integral and important roles in biofilm development, contributing to both periodontal health and disease (Kolenbrander et al., 2010). Due to its elongated shape and adhesin production, it acts as a bridge-organism by connecting microorganisms and cells (Kaplan et al., 2009; Wu et al., 2015). *F. nucleatum* possesses a mutualistic relationship with other members of the oral microbiota, and its interactions with human tissues range from neutral to pathological interactions. In the particular case of periodontitis, *F. nucleatum* increases the infectivity of other pathogenic oral microorganisms, thereby underpinning this disease (Brennan and Garrett, 2019). In particular, *F. nucleatum* may induce expression of the β-defensin 2 peptide and certain pro-inflammatory cytokines (Krisanaprakornkit et al., 2000; Brennan and Garrett, 2019) as well as increase the invasive potential of *Porphyromonas gingivalis* (Taxman et al., 2012), suggesting that during periodontitis these bacteria act cooperatively to evade the immune system and develop an inflammatory-permissive environment.

Indeed, studies have shown that *F. nucleatum* is involved not only in oral inflammation such as periodontitis (Krisanaprakornkit et al., 2000), but also in brain abscesses (Kai et al., 2008), pericarditis (Han et al., 2003), Lemierre syndrome (Weeks et al., 2010), and in acute appendicitis (Swidsinski et al., 2011). The microbiome analyses of colorectal carcinomas reveal a significant enrichment of *Fusobacterium* species, in particular phylotypes more similar to *F. nucleatum*, *Fusobacterium mortiferum* and *Fusobacterium necrophorum*. The enrichment of the fusobacterial load in cancer is confirmed by histological analysis on tumor tissues when compared to adjacent tissues and by the DNA of *F. nucleatum* found in the CRC metastases (Kostic et al., 2012). Moreover, it has been demonstrated that patients with CRC possess identical strains of *F. nucleatum* in their CRC and saliva specimens. Although the relationship between these bacteria and CRC is not well-understood, this finding does suggest that *F. nucleatum* strains associated with CRC may have originated from the oral cavity (Komiya et al., 2019). High abundances of these bacteria are also found at the level of adenomas, epithelial neoplastic lesions that can become malignant and be precursors of most colorectal cancers. In fact, in patients with early CRC (Yachida et al., 2019) *F. nucleatum* increases in abundance during the very early stages of carcinogenesis, thus suggesting that it could be involved in the tumor onset and progression (Kostic et al., 2013). Its abundance in colorectal instead of adjacent tissues may be caused by the strong adhesive and invasive abilities of fusobacteria toward colonic epithelial cells due to the *FadA* surface protein (*Fusobacterium* adhesin A), which interacts with E-cadherin to mediate changes in β-catenin and other signaling pathways, thereby inducing inflammatory changes and contributing to carcinogenesis (Han et al., 2000; Strauss et al., 2011; Rubinstein et al., 2013). The presence of *F. nucleatum* cells plays an important role also on the binding between Gal-GalNAc and Fap2, becoming overexpressed in CRC (Abed et al., 2016). The high numerical presence of fusobacteria at the tumor site may also be derived from the growth advantage that fusobacteria provide to the tumor by eliciting myeloid immune cell responses that promote tumor growth. Fusobacteria elicit a metabolic advantage to tumor cells in a competitive tumor environment. Like non-saccharolytic bacteria and in contrast to *Enterobacteriaceae*, fusobacteria, which can metabolize glucose and amino acids, will not compete for glucose in a tumor microenvironment as they can use amino acids and peptides as nutrient sources, thereby supporting tumor metabolism (Vander et al., 2009). *F. nucleatum* strains can by means of a rudimentary electron transport chain establish a respiratory-like metabolism (Kapatral et al., 2002) allowing them to persist and slowly replicate in the hypoxic tumor microenvironment. Due to its ability to express adhesive molecules, *F. nucleatum* is able to form biofilms that enhance oxygen tolerance (Gursoy et al., 2010). A previous study has shown that the administration of *F. nucleatum* in ApcMin/+ mice, which carry a point mutation in the Adenomatous Polyposis Coli (APC) gene on a single allele, accelerates the progression and carcinogenesis, provokes infiltration of specific myeloid cells into the tumor and creates a pro-inflammatory environment through the induction of the NF-κB pathway (Kostic et al., 2013). This suggests that the tumorigenic effects of fusobacteria operate downstream of the loss of the APC tumor suppressor and the consequent intestinal dysplasia that occurs in ApcMin/+ mice. Moreover, this may explain why increased abundance of *F. nucleatum* occurs already during the first phase of adenoma, as the APC mutation is among the first molecular alterations that arise in the epithelium while it is in transition to become adenoma (Kostic et al., 2013). However, early somatic mutations that can lead to loss of tight junction, cellular contacts, polarity and mucus layer in the gut, may promote infiltration and enrichment of *Fusobacterium* spp.

Furthermore, *F. nucleatum* may function as a predictive marker of tumor recurrence because its numbers are increased in CRC patients who show post-chemotherapy relapse (Sun et al., 2019). It was therefore assumed that it may induce chemoresistance by blocking chemotherapy-induced apoptosis and activating pathway for autophagy by inducing upregulation of multiple autophagy signaling elements (pULK1, ULK1, and ATG7) (Yu et al., 2017).

The Fusobacteriales order also includes the *Leptotrichiaceae* family, whose *Leptotrichia* spp. appears to be predominantly present in CRC tissues (Warren et al., 2013). *Leptotrichia* spp. are Gram-negative facultative anaerobes, commensal members of the oral microbiome and subgingival plaque, but they can also be present in the gut, urogenital system, and female genital tract (Eribe et al., 2004). Isolated from several periodontal lesions, abscesses and systemic infections, they are opportunistic pathogens (Manson et al., 2014). In fact, previous studies describe the occurrence of *Leptotrichia buccalis*-mediated bacteremia in patients with neutropenia and progressive malignancy, though its incidence in serious bacteremic infections remains comparatively low (Reig et al., 1985; Weinberger et al., 1991).

Due to its microbial co-aggregation ability, *Fusobacterium* is found together not only with *Leptotrichia* but also with *Campylobacter* spp., which are all anaerobic bacteria that commonly colonize the same niche in the oral cavity. Co-occurrence of *Fusobacterium* and *Campylobacter* spp. is observed in CRC patients with a prevalence of *Campylobacter* spp. in CRC lesions compared with adjacent healthy tissues (Warren et al., 2013). Some genotoxins produced by enteric pathogenic species such as *Salmonella*, *Escherichia*, and *Campylobacter* have a synergistic effect on carcinogenesis (Guerra et al., 2011; Bezine et al., 2014). *Campylobacter jejuni* is a well-characterized human pathogen and one of the main causes of acute gastroenteritis and colitis (Brauner et al., 2010). A specific cytolethal distending toxin (CDT), produced by *C. jejuni* and composed of three subunits, plays a key role in carcinogenesis. In particular, one specific subunit (CdtB) is implicated in promoting carcinogenesis, since mutation of the corresponding gene in *C. jejuni* causes reduction of both tumor cells and neoplastic progression. This subunit, which can induce extensive DNA damage in host cells and in turn provoke cell apoptosis, is known to stimulate tumor proliferation (He et al., 2019). The above mentioned CDT activities have previously been associated with carcinogenesis in studies reporting high level of *cdt* mutant strains present both in biopsies of CRC patients and in hepatocarcinogenesis and intestinal tumorigenesis in mice (Ge et al., 2007; Buc et al., 2013; Ge et al., 2017).

However, the precise role played by *Fusobacterium, Leptotrichia*, and *Campylobacter* in the etiology of carcinogenesis is still not fully understood and requires further study.

## New Diagnostic Approaches Using Microbial Markers

A lot of effort has been devoted to identifying microbes that can be employed as biological markers for CRC. At the same time, research endeavors have also focused on the most appropriate technique to detect such microbes. The most frequently used method is a test performed on the sera of CRC patients, allowing the detection of cell-free DNA (cfDNA) corresponding to DNA fragments originating from tumor cells. These fragments can be further examined for mutations and genomic abnormalities, providing both diagnostic and prognostic biomarkers (Tan et al., 2016). So far, different diagnostic tests, being useful for the detection of CRC but having low specificity and sensitivity, are performed on fecal occult blood. Additionally, the detection of mutated DNA in stool is a promising technique (Dhaliwal et al., 2015). Detection of IgA and IgG antibodies against some of these potential microbial biomarkers may also represent a future diagnostic tool (Wang et al., 2016).

Various recent studies involving large patient cohorts have focused on the characterization of the microbiome of colorectal adenomas and on cancer, aimed at assessing the presence of *F. nucleatum* in stool and tissue samples of patients, who had received a positive CRC diagnosis (Kostic et al., 2012; McCoy et al., 2013; Peng et al., 2018).

Diagnostic tests designed to determine the presence of biomarkers are very useful not only for diagnosis, but also for assessing the patient’s prognosis and for developing therapies aimed at combating the onset and progression of tumors. However, there are ongoing debates on the use of *F. nucleatum* as a reliable CRC biomarker. Not only the method for microbial detection is important in this context, but also the predictive accuracy of microbial biomarker(s) using larger population-scale studies that also take into account the differences due to ethnicity and geography (Brennan and Garrett, 2019).

As *F. nucleatum* appears to influence myeloid cell tumor infiltration, the phenotype of T cells and the cytotoxic activity of NK cells has received significant scientific attention (Routy et al., 2018). Furthermore, the high abundance of *F. nucleatum* in tissues and fecal samples of cancer patients who show relapse after having undergone chemotherapy is indicative of the impact of this bacterium on chemotherapy resistance. This potential biomarker activates the process of autophagy and compromises chemotherapy-mediated cancer cell death (Yu et al., 2017). These findings have resulted in a therapy that is directed to specifically target *F. nucleatum*, before or concomitant with the administration of chemotherapy. Most *F. nucleatum* isolates are sensitive to antibiotics such as erythromycin, other macrolides (Riordan, 2007), metronidazole (Bullman et al., 2017) and numerous β-lactam based antibiotics with the exception of penicillin (Nyfors et al., 2003). Additionally, epidemiological and clinical data suggest that non-steroidal anti-inflammatory drugs (NSAIDs) such as aspirin may be effective as a primary and secondary preventive measure in CRC (Chan et al., 2012). However, it may be more advantageous to use narrow-spectrum antibiotics specific for *F. nucleatum* and targeting tumor tissue in order to protect the anaerobic bacteria that play a crucial role in improving the response to chemotherapy and immunotherapy (Brennan and Garrett, 2019).

Currently, cancer treatment strategies are increasingly focusing on immunotherapy and chemoprevention. The former one, including the use of COX-2 inhibitors and selective EP2 antagonists, plays a significant role in counteracting *F. nucleatum*-associated CRC (Shang and Liu, 2018). EP2 enhances the expression of NF-κB-targeted proinflammatory genes induced by TNF-α in neutrophils, promoting colon tumorigenesis by means of expanding inflammation and creating a tumor microenvironment. Selective EP2 antagonists are promising drugs for the chemoprevention of *F. nucleatum*-associated CRC (Ma et al., 2015). COX-2 is considered an inhibitor of antigen-specific tumor immunotherapy. Therefore, COX-2 inhibitors reduce the risk of CRC by inhibiting inflammatory pathways, and the use of such inhibitors may therefore be important to enhance efficacy of immune-based therapy in CRC patients (Gobel et al., 2014). Immunotherapy may also represent an effective strategy to prevent *F. nucleatum*-positive CRC. The interaction between Fap2 and TIGIT receptor protects tumors against immune cell attack and inhibits antitumor immunity (Gur et al., 2015).

The reduction of *Fusobacterium* populations in the oral cavity, where they are most abundant, or in the gastrointestinal tract may work to delay or prevent tumor progression for patients at increased risk of CRC (Kostic et al., 2013). For this reason, *F. nucleatum* has been the target of vaccine and/or antimicrobial therapies. The formulation of a possible vaccine has already been tested to fight the problem of halitosis. This vaccine targets FomA, which is a protein of the outer membrane expressed by certain strains of *F. nucleatum* and necessary for bacterial co-aggregation and its associated pathogenicity. Inhibition of co-aggregation by inactivation of *F. nucleatum* FomA will prevent the progress of oral infections (Liu et al., 2010). Another option to reduce *F. nucleatum* abundance could be a replacement therapy of the microbial ecosystem aimed at modifying host and tumor microbiota through the use of consortia of engineered microorganisms or selected cocktails of human-derived isolates (Petrof et al., 2013).

## Immunotherapy as a New Frontier in the Fight Against Cancer

Cancer remains a major cause of mortality and many of the therapies that have been used so far to fight it are very often ineffective and bring high degree of toxicity (Puzanov et al., 2017). Until recently, cancer was routinely treated through surgical, chemotherapy and/or radiotherapy approaches (Yu W.D. et al., 2019). However, the high level of toxicity and the high incidence of cancer recurrence always make these therapies desirable or effective. A new frontier in cancer therapy is represented by immunotherapy (Yang, 2015), which holds a lot of promise in terms of therapeutic success and allows tumor targeting in a much more specific way than other currently applied therapies. In addition, immunotherapy offers the advantage of immune system memory against malignant cells to achieve a durable cure with minimal toxicity (Helmy et al., 2013). Two forms of immunotherapy are currently recognized: (i) “passive” immunotherapy that includes agents such as cytokines, antibodies and transferred immune cells that target the tumor directly, and (ii) “active” immunotherapy that mobilizes the immune system to eliminate the tumor (through vaccination) (Finn, 2012).

The antibody-mediated approach is a well-established, specific immunotherapy for cancer in clinical practice (Finn, 2012). Monoclonal antibodies for therapeutic purposes have been designed to bind with high affinity to specific cell surface molecules on cancer cells to direct the immune system toward the elimination of malignant cells (Shepard et al., 2017).

Perhaps the most intriguing class of antibody therapeutics currently being developed for cancer includes the one designed to activate anti-tumor therapeutic immunity, encompassing the immune checkpoint blockade (ICB). Immune checkpoint therapy targets regulatory pathways in T cells by removing their inhibitory signals, thereby enabling tumor-reactive T cells to overcome regulatory mechanisms and to mount an effective antitumor response (Sharma and Allison, 2015). Since many of these antibodies are activated by ligand-receptor interaction, the immune checkpoints can be readily blocked by antibodies or modulated by recombinant forms of ligands or receptors (Routy et al., 2018). The two most actively studied are cytotoxic T-lymphocyte-associated antigen 4 (CTLA-4) and programmed cell death protein 1 (PD-1), which are both inhibitory receptors, regulating immune responses at different levels and by different mechanisms (Pardoll, 2012). In 2011, CTLA-4 became the first validated target of ICB in the treatment of patients with melanoma, and in spite of the beneficial effects, this therapy is accompanied by various toxic effects that can sometimes lead to autoimmune issues (Prieto et al., 2012; Schachter et al., 2017).

In the same manner, different antibodies that disrupt the interaction between PD-1 and its ligands have been approved for therapeutic purposes. PD-L1 together with PD-L2 are binding and functional partners of PD-1, expressed on the surface of many organ cells and in various tissues (Freeman et al., 2000; Latchman et al., 2001) playing a dominant role in the suppression of T cell responses, especially in the tumor microenvironment (Zou and Chen, 2008), thereby preventing effector immune cells from killing cancer cells (Azuma et al., 2008). Recent studies have shown how anti-PD-1 outperforms anti-CTLA-4 therapy in efficacy, survival and adverse events (Schachter et al., 2017).

A novel way to fight cancer employs genetic engineering of the immune effector cells in order to modify their functions. Following the interest given to the first successful treatments with chimeric antigen receptor (CAR) T-cell in B-cell acute lymphoblastic leukemia (Maude et al., 2018), high level expectations have been created which will require detailed investigations (Kochenderfer et al., 2010; Kalos et al., 2011). CAR T-cells are produced by transducing a genetically engineered CAR fusion protein by means of a retrovirus or lentivirus into autologous T-cells (Sermer and Brentjens, 2019).

However, ICB and CAR T-cell therapies are not universally effective due to the genetic instability of tumors which may lead to cessation of the expression of antigens targeted by T cells or which may eliminate the mechanisms that present them (Bronte and Mocellin, 2009).

## Bacteria Eliciting Beneficial Effects Toward Cancer Targeted by Immunotherapies

ICB has revolutionized the therapeutic approach in immunogenic cancers like melanoma (Vetizou et al., 2015) and renal cell carcinoma (RCC) (Motzer et al., 2015) as well as malignancy considered non-immunogenic like non-small cell lung cancers (NSCLC) (Borghaei et al., 2015; Carbone et al., 2017) or mismatch-repair-deficient colorectal cancer (Le et al., 2015).

**FIGURE 1:**
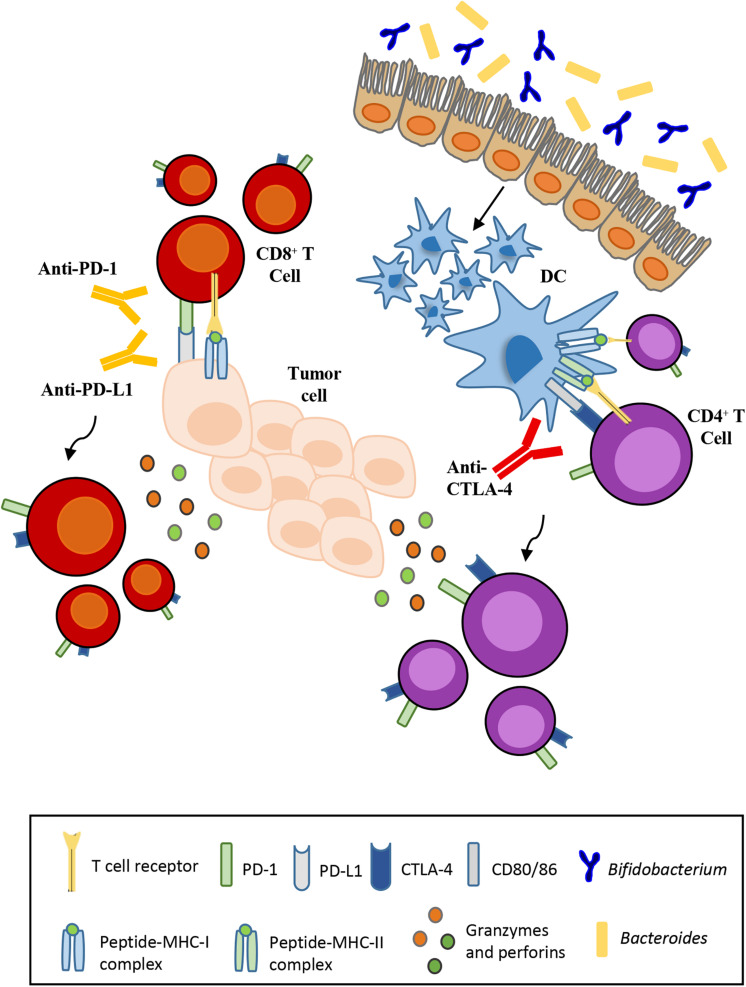
Influence of the gut microbiota on the effectiveness of immunotherapy. Anti-CTLA-4 and anti-PD-L1 therapies depend respectively on *Bacteroides* enrichment and *Bifidobacterium* abundance for their efficacy. T-cell activation and proliferation against tumor cells requires monoclonal antibodies that block the interaction between ligand and its respective receptor.

Various studies have indicated that microbiota composition impacts on the efficacy of ICB therapies. The use of antibiotics to induce intestinal dysbiosis in preclinical mouse models has underlined the contribution of certain commensal bacteria such as *Bacteroides fragilis* (Vetizou et al., 2015) and *Bifidobacterium* (Sivan et al., 2015). Mice treated with broad-spectrum antibiotics or germ-free (GF) mice that lack some bacterial species, in particular *Bacteroides*, are resistant to CTLA-4 blockade therapy. The response to the inhibition of CTLA-4 is regained with the oral administration of *Ba. fragilis*. Recolonization of the intestinal microbiota by *Ba. fragilis* consequently causes T-cell helper (TH1) responses to increase in the lymph nodes closest to the tumor, thereby improving the efficacy of the CTLA-4 blockade. A similar significant response is observed in cases of fecal transplantation of *Bacteroides* species in GF mice (Figure 1; Vetizou et al., 2015). In parallel, another trial compared the antitumor cytotoxic T lymphocytes (CTLs) responses in mice purchased from two different facilities differing in their commensal microbes. Indeed, Jackson Laboratory (JAX) mice but not Taconic Farms (TAC) mice, may be colonized by commensal microbes that facilitate antitumor immunity. Of note, *Bifidobacterium* was found to be particularly abundant in the colon of JAX mice that exhibited reduced growth of melanomas and improved CTL-mediated immune-surveillance. The presence of *Bifidobacterium* was shown to be positively associated with antitumor T cell responses, indicating that certain species of this genus, identified as *Bifidobacterium breve*, *Bifidobacterium longum* and *Bifidobacterium adolescentis*, elicit beneficial antitumor immune effects (Sivan et al., 2015).

In addition, the selective transfer of *B. breve* or *B. longum* into mice that typically are devoid of these species was sufficient to reduce melanoma natural growth and restore anti-melanoma specific T cell responses. As a result, the frequency of tumor-specific CTLs residing in melanoma lesions increased in mice carrying *B. breve* or *B. longum* cells, on respect of germ-free mice or mice without bifidobacteria in the gut (Figure 1; Sivan et al., 2015). The use of bioluminescent imaging (BLI) allows the detection of certain bacterial species, including species of bifidobacteria, following administration in tumor-bearing mice (Cronin et al., 2012). Preclinical therapeutic studies had already demonstrated the ability of different bacterial strains to migrate to the tumor site (Grillot-Courvalin et al., 1998). Once administered, bifidobacteria can survive in the hypoxic tumor environment due to the nutrient-rich environment created by cell death in necrotic regions. This finding demonstrates the potential for non-pathogenic bacteria as vectors for cancer therapy in order to deliver therapeutic or diagnostic agents (Cronin et al., 2012).

The specific mechanism by which bifidobacteria or other commensal bacteria stimulate antitumor immune responses remains to be elucidated. However, it has been shown that these bacteria stimulate the maturation of dendritic cells that, like antigen-presenting cells (APC), play a role in activating T-cells. CTLA-4 is a homolog of APCs’ receptor that binds with higher affinity and downregulates T-cell activation. Anti-CTLA-4 monoclonal antibodies block this interaction favoring T-cell activation and proliferation (Krummel and Allison, 1995). In contrast, PD-1 has two ligands, i.e., PD-L1 and PD-L2, where PD-L1 is expressed by cancer cells and tumor-infiltrating macrophages, while PD-L2 is expressed by APCs (Francisco et al., 2010). The interaction of PD-L1 with PD-1 may induce T cell suppression. PD-1 blockade by monoclonal antibodies restores the function of T-lymphocytes.

The translational relevance of these findings to humans was then shown in other studies that clearly demonstrated the significant contribution of different commensals in the positive response to immunotherapy treatment against different types of cancer; *Akkermansia muciniphila* on NSCLC or RCC patients, and *Fecalibacterium* spp. or *Bifidobacterium* spp. on melanoma patients (Gopalakrishnan et al., 2018b; Matson et al., 2018; Routy et al., 2018).

Recently, the impact of antibiotics (ATB) use in patients with different types of cancer (lung, renal, urothelial) who were treated with PD-1/PDL-1 inhibitors was investigated (Routy et al., 2018). As observed in murine-based trials, patients treated with ATB show reduced survival regardless of the type of tumor and a general reduction in the anti-PD-1/PD-L1 therapeutic responses. From the analysis and comparison of the microbiota obtained from fecal samples of the immunotherapy responding (R) and non-responding (NR) subjects (according to the best clinical response as assessed by Response Evaluation Criteria in Solid Tumors), differences were particularly noted in the abundance of *A. muciniphila*, which was more present in R patients and positively associated with an increase of more than 3 months of tumor-free survival (Routy et al., 2018). An increased abundance of other commensals such as *Ruminococcus* spp., *Alistipes* spp. and *Eubacterium* spp., was also observed, while *B. adolescentis*, *B. longum*, and *Parabacteroides distasonis* were underrepresented (Matson et al., 2018). To test the effective correlation between *A. muciniphila* and the response to PD-1/PDL-1 inhibitors, a recolonization of ATB-treated mice reared in specific pathogen-free (SPF) conditions (or alternatively GF animals) by fecal microbiota transplantation (FMT) was performed using patient stool by oral gavage of feces harvested at diagnosis from different NSCLC patients, R and NR. This *in vivo* test corroborated the clinical data according to which mice receiving FMT from R, therefore with marked presence of *A. muciniphila*, demonstrated a better response to immuno-oncological therapies (Routy et al., 2018) and a significant reduction in tumor size with a greater accumulation of immune cells at the level of the cancerous microenvironment. Indeed, the release of IL-12 cytokines, which support the role of T lymphocytes, in response to the significant presence of *A. muciniphila* (Sivan et al., 2015), seems to have increased. However, the precise immunomodulatory mechanism still remains unclear (Collado et al., 2007). Moreover, the clinical significance of the gut microbiota as a novel biomarker of immune checkpoint inhibitor (ICI) response needs to be validated in prospective studies.

Evaluation of the gut microbiota composition of patients with cutaneous melanoma treated with anti-PD-1 confirmed a marked presence of Clostridiales and Ruminococcaceae bacteria, especially *Faecalibacterium* in the intestine of R patients, while in NR patients *Bacteroides thetaiotaomicron*, *E. coli*, and *Anaerotruncus colihominis* are more abundant (Gopalakrishnan et al., 2018b). High abundance of *Faecalibacterium* was positively correlated with a significantly prolonged progression-free survival, in line with recently published data (Chaput et al., 2017). It is worth mentioning that these dissimilar microbial compositions observed in different studies may be due to the use of different models or analytical methodologies, for example the use of mice as opposed to human beings. In the latter case, as already mentioned, age, diet and geographical position also influence the intestinal bacterial composition. In addition, the specific anticancer drug used in the immunotherapy approach and the different type of malignant tumor are likely to have a key effect on the microbial diversity found.

## Bifidobacterial Immunomodulatory Effects

As discussed above, modification of the gut microbiota appears to provide a novel way to improve the efficacy and reduce the side effects of current anticancer therapeutic approaches (Villeger et al., 2019). Many strategies are considered to enhance the effectiveness of cancer treatment, such as modulation of the intestinal microbiota, which is currently receiving a lot of scientific attention (Bashiardes et al., 2017; Helmink et al., 2019). The use of microorganisms known as probiotics, i.e., microbes which, when administered in adequate amounts, confer health benefits to the host, is becoming an important research field (Gibson et al., 2017). There are several beneficial effects of probiotics on host health, from blocking pathogenic bacteria to promoting intestinal epithelial cell survival, but the most important is the modulation of the immune system (Yan and Polk, 2011). Bifidobacteria are among those bacteria that are currently widely used as probiotics and that are capable of interacting with the immune system (Villeger et al., 2019). A growing number of studies have highlighted bifidobacteria as commensal organisms capable of stimulating and modulating specific pathways, through which they influence the host immune responses, both innate and adaptive (Palmer et al., 2007; Arboleya et al., 2016; Hidalgo-Cantabrana et al., 2017; Pickard et al., 2017; Ruiz et al., 2017; Alessandri et al., 2019). In fact, various strains of *Bifidobacterium* individually or in combination with other strains have been evaluated as probiotics for different diseases and some of these have shown quite promising results in alleviating the symptoms of IBD, IBS, diarrhoa and allergy (Tojo et al., 2014). However, the molecular mechanisms underlying the interaction between bifidobacteria and the host immune system are not yet fully understood.

First described in 1899, bifidobacteria are Gram-positive, anaerobic, non-motile, non-sporulating, saccharolytic, and bifid-shaped microorganisms with a high G + C DNA content (Ventura et al., 2007). Beyond their carbohydrate metabolism functions (Milani et al., 2015), bifidobacteria are widely exploited by food and pharmaceutical companies as health-promoting microorganisms (Linares et al., 2017). The molecular mechanisms, by which these bacteria colonize the intestine, adhere to the host’s intestinal epithelium and elicit a positive effect on the immune response, represent a current and active research topic. There are some extracellular structures, secreted enzymes and bioactive metabolites that have been implicated to play a fundamental role in the interaction of bifidobacteria with their hosts (Turroni et al., 2013; McCarville et al., 2017; Alessandri et al., 2019; O’Connell Motherway et al., 2019). In the following section, some salient details of these extracellular structures identified in bifidobacteria are discussed.

### Exopolysaccharides

The cell envelope of a wide range of bacteria is covered by one or more glycan layers known as capsular polysaccharides (CPS) or exopolysaccharides (EPS). From a research point of view EPS producers have received substantial interest as these extracellular polymers have been reported to play a specific role in host-microbe interactions and human health by promoting adhesion to the intestinal mucosa, as well as by modulating the intestinal microbiota composition, and conferring selective advantage to bacteria through protection to adverse conditions such as presence of bile salts or pH insults (Fanning et al., 2012a). For example, *Bifidobacterium animalis* subsp. *lactis* has developed strategies to tolerate physiological bile salt concentrations by synthetizing EPS, probably as a mechanism of protection against toxic compound (Ruas-Madiedo et al., 2009).

Some of these microbial biopolymers are also receiving renewed interest due to their involvement in promoting human health (Ruas-Madiedo et al., 2009; Ferrario et al., 2016). In this context, an *in vitro* experiment was carried out to evaluate the level of stimulation of the pro and anti-inflammatory cytokines following contact with the EPS extracted from different bifidobacterial species. This study revealed that the differentiation of T cells is strongly influenced by the physical-chemical features of the particular EPS used. Two different *B. adolescentis* strains (IF1-03 and IF1-11) not only stimulate the production of anti-inflammatory cytokines but also contribute to the reduction of the area of ulceration and thickening of the intestinal wall (Yu R. et al., 2019). In addition, a recent *in vivo* study reported that a *Bifidobacterium bifidum* strain due to the presence of a cell surface-associated β-glucan/galactan (CSGG) can induce the generation of Foxp3^+^ regulatory T cell, eliciting a strong suppressive activity toward experimental colitis (Verma et al., 2018).

This finding suggests that a positive correlation exists between the composition, structure and size of a given EPS polymer and the corresponding elicited immune response (Salazar et al., 2014). Similar results were obtained in other *in vitro* studies, which were confirmed by *in vivo* trials (Hidalgo-Cantabrana et al., 2014; Yu R. et al., 2019). In this context, it has been demonstrated that the EPS-producing *B. breve* UCC2003 strain evokes lower expression of proinflammatory cytokines interferon alpha (IFN-α), TNF-α, and IL-12 in splenocytes isolated from naïve mice and this finding suggests that the EPS layer plays a crucial role in the persistence of this strain in the host intestine, reducing the risk of immune clearance against this microbial strain (Fanning et al., 2012a). Notably, the genome of *B. breve* UCC2003 has been shown to encompass two putative EPS-encoding clusters. One cluster (*epsRhm*) was found to include genes that are putatively responsible for rhamnose biosynthesis, whereas the second cluster (*eps*) presents two adjacent oppositely oriented genes (*eps*1 and *eps*2), encodes regulatory components, glycosyltransferases and export functions (Fanning et al., 2012b). According to previous studies, *B. breve* UCC2003 EPS, metabolized by members of the infant microbiota, promotes the health status of infants (Pungel et al., 2020) and downregulates apoptotic responses to protect epithelial cells under imposed inflammatory conditions (Hughes et al., 2017), supporting the notion that EPS-mediated immune response is influenced by the physicochemical nature of these polymers.

### Pili/Fimbriae

Pili or fimbriae are proteinaceous extracellular appendages produced by many bacteria, that protrude from the bacterial cell surface and that can be involved in microbe-host interactions promoting adhesion to the intestinal epithelium or facilitating aggregation with other bacterial cells (Scott and Zahner, 2006; Kline et al., 2010; Foroni et al., 2011). Two different types of pili have been described in bifidobacteria, i.e., sortase-dependent pili, and the type IVb pili, both of which are also known as tight adherence pili (Tad pili) (O’Connell Motherway et al., 2011; Milani et al., 2017b). Bifidobacterial sortase-dependent pili are not only responsible for adhesion and interaction with the host, but also are involved in the microbe-microbe interactions and in stimulation/modulation of the host immune system. Indeed, a case study focused on *B. bifidum* PRL2010 demonstrated that sortase-dependent pili have a crucial role in promoting aggregation between bacterial cells of a heterogeneous population, increasing the colonization of host intestinal mucosa (Turroni et al., 2014). Similarly, a related study highlighted that sortase-dependent pili produced by *B. bifidum* PRL2010 activated various signals in macrophages by locally inducing high levels of the cytokine TNF-α, yet reducing the expression of other pro-inflammatory cytokines, such as IL-12, associated with systemic response (Turroni et al., 2013). Apparently, this facilitates cross-talk between this bifidobacterial strain and host immune cells without causing a detrimental inflammatory cascade response.

The other bifidobacterial pilus type, the Tad pilus, that has been characterized in detail in the model organism *B. breve* UCC2003 (O’Connell Motherway et al., 2011, 2019; Milani et al., 2017b), has been shown to promote the maturation of epithelial cells, stimulating growth of their immature intestinal mucosa and contributing to host mucosal homeostasis (O’Connell Motherway et al., 2019). However, this is still a hypothesis that has not been proven yet in humans though demonstrated in murine models.

### Serpins

Serpins (Serine protease inhibitors) are prokaryotic and eukaryotic enzymes, synthetized by particular members of the bifidobacterial intestinal community and involved in the regulation of various protease-mediated processes (Potempa et al., 1994; Turroni et al., 2010). The production of serpins is not widespread in bifidobacteria, in fact it has been identified only in few species like *B. breve*, *B. longum* subsp. *longum*, *B. longum* subsp. *infantis*, *B. longum* subsp. *suis*, *Bifidobacterium cuniculi*, *Bifidobacterium scardovii*, and *Bifidobacterium dentium* (Turroni et al., 2010). Notably, the expression of serpin-encoding genes is induced in response of the presence of a specific two-component regulatory system (Alvarez-Martin et al., 2012). Bacterial infection or intestinal tissue damage typical of inflammatory bowel diseases and ulcerative colitis are the main factors by which serine proteases may be released. Beyond eliciting anti-inflammatory activity through the prevention of negative effects of high levels of human serine proteases, serpins may assist bifidobacteria to protect themselves against host-derived proteases and survive in a competitive environment (Turroni et al., 2010; Kainulainen et al., 2013). Recently the anti-inflammatory efficacy of these enzymes has been demonstrated in the prevention of gluten-related immunopathology, of which effects of are significantly alleviated due to the ability of serpin to modulate the immune system, to maintain barrier function and to inhibit elastases released during inflammation (McCarville et al., 2017).

Besides pili, EPS and serpins, there are other bifidobacterial-associated extracellular proteins affecting the host immune system. A specific *B. bifidum* strain is able to produce two type of extracellular molecules such as BopA and TagA. The latter is a protein located on the outer bacterial surface that acts like a peptidoglycan lytic enzyme causing the activation or the proliferation of dendritic cells and the induction of IL-2 (Guglielmetti et al., 2014). BopA is a surface-associated protein not only able to stimulate production of IL-8 but also to enhance adhesion of bifidobacteria to epithelial cells through the high hydrophobicity of this lipoprotein (Guglielmetti et al., 2008; Kainulainen et al., 2013).

Finally, bifidobacterial metabolism influences intestinal immune homeostasis and inflammatory response through microbe-microbe cross-feeding activities (Alessandri et al., 2019). Bifidobacterial metabolism of non-digestible carbohydrates leads to the production of acetate and lactate, which in turn can be converted by secondary degraders into butyrate, thereby resulting in a so-called butyrogenic effect (O’Callaghan and van Sinderen, 2016). Various studies have reported on the mutual beneficial effects of co-cultivation of *Bifidobacterium* strains with butyrate producers in the presence of diet-derived sugars and host-derived glycans promoting growth yield of both strains (Rios-Covian et al., 2015; Riviere et al., 2015; Schwab et al., 2017; Bunesova et al., 2018). Moreover, bifidobacteria, unlike other enteric microorganisms such as *Bacteroides*, display a limited hydrolytic capacity toward xylan (Ejby et al., 2013). In fact, bifidobacteria are not able to grow on xylan on their own, nevertheless they manage to grow on this substrate when co-cultivated with *Bacteroides ovatus*. This phenomenon is due to the extracellular activity of *Ba. ovatus* that degrades xylan chains, allowing an efficient uptake of the produced xylo-oligosaccharides by a dedicated ABC transporter encoded by various bifidobacterial species (Rogowski et al., 2015).

## Bacterial Therapy Supporting Immunotherapies

The complexity of the gut microbiota plays a key role in the response to the ICI. Therefore, the benefit of the treatments are reduced in those patients who have taken antibiotics and thus display an intestinal microbiota of reduced diversity (Villeger et al., 2019). Moreover, patients responding to the therapy have a different microbiota, in species composition and diversity, compared to patients that do not respond to immunotherapy (Routy et al., 2018). As mentioned above, accumulating evidence suggests that modulation of the gut microbiota affects the host responses to various forms of cancer therapy, most notably immunotherapies (Robertson et al., 2017). Several methods are currently being studied including the use of prebiotics, probiotics and fecal microbiota transplantation. The notion of using microbial components or their products in anti-cancer therapy dates back to 1891 when Coley used killed *Streptococcus pyogenes* in combination with a second killed organism now known as *Serratia marcescens* in the treatment of bone sarcoma (McCarthy, 2006). 16S ribosomal RNA (rRNA)-based sequencing of gene amplicons and shotgun metagenomics analyses of stool samples allow the identification of particular bacteria that are more abundant in responding vs. non-responding patients (Matson et al., 2018; Elkrief et al., 2019). Moreover, researchers have identified a consortium of human-associated bacterial strains acting together to induce interferon-γ-producing CD8 T cells in order to confer resistance to certain bacterial infections, such as *Listeria monocytogenes*, while also being effective in inhibiting tumor growth in conjunction with ICIs (Tanoue et al., 2019). These findings reinforce the notion that the gut microbiota can be considered as a therapeutic target in the treatment of various diseases through manipulation of host physiological functions, which may be associated with less risk when compared to other biotherapeutic approaches (Tanoue et al., 2019). Data supporting the important role for improved immunotherapeutic efficacy have been obtained by transferring fecal bacteria from responsive patients into GF or antibiotic-treated SPF mice, which has been inoculated with tumors and treated with mAbs to CTLA-4 or PD-1/PD-L1 (Vetizou et al., 2015). However, there are several critical parameters to consider for this approach. For example, fecal material should be sourced from a healthy individual who has been screened in order to eliminate the risk of inadvertently transmitting infections that could cause inflammation-induced carcinogenesis or formation of dysplasia or polyps (Wong et al., 2017; Chen et al., 2019; Fessler et al., 2019). FMT is a biological drug recognized by the U.S. Food and Drug Administration, though its safety remains a controversial issue because of the unidentified composition and pathogenicity of fecal bacteria that might be transmitted (Chen et al., 2019).

Another means of intervention may be modulation of the autochthonous commensal microbial community via prebiotics or dietary changes to favor colonization and expansion of selected beneficial bacteria (Zitvogel et al., 2018). A prebiotic is defined as a selectively fermented ingredient that allows specific changes, both in the composition and/or activity in the gastrointestinal microbiota that confers benefits upon host well-being and health (Roberfroid, 2007). For example it has been shown that they could favor the proliferation of beneficial bacterial species that are already present in the host, such as *Faecalibacterium*, *Eubacterium*, and *Roseburia* spp. These taxa are also able to produce organic acids (i.e., produce acetate, propionate and butyrate), that are known to play a role in preventing cancer and may have both local and systemic biological effects; in particular butyrate, a preferred energy source of colonocytes (Ambalam et al., 2016). Even probiotics may possess anticancer effects at different stages of carcinogenesis, being attributable to the binding of mutagens or carcinogens, with subsequent biotransformation into less toxic metabolites (Raman et al., 2013). *In vivo* studies have provided evidence that administration of probiotics has significant protective effects against CRC by reducing aberrant crypt foci (ACF), producing SCFA, down-regulating pro-inflammatory cytokines, inhibiting pathogens and cancer-causing microbes, and by immune-stimulation and reduction of pro-carcinogenic enzymatic activities (O’Mahony et al., 2001; Bertkova et al., 2010; Pithva et al., 2015). In this context, fermented products are known to be an important source of both nutrients and microorganisms. Microbial metabolites and live microorganisms are considered to have positive effects on host health and in this context there is robust evidence that the intake of fermented foods significantly decreases cancer risk, bladder cancer, CRC and esophageal cancer risk. In contrast, the intake of fermented foods is inversely correlated with prostate cancer, renal cancer and ovarian cancer risks (Zhang et al., 2019). The precise mechanisms involved have not yet been described and further studies should be done to confirm such preliminary yet exciting results. Recently, research has revealed the critical role played by CD47, which is a widely expressed protein present on the surface of many cancer cells triggering a deleterious signal to the macrophages inviting them not to attack (Advani et al., 2018). Experiments have shown that tumor-bearing mice, which are normally respondent to anti-CD47 treatments, failed to obtain benefits from therapy when intestinal bacteria were destroyed by taking a cocktail of antibiotics. In contrast, mice that did not respond to immunotherapy were shown to benefit from cancer treatments when they were receiving a mixture of *Bifidobacterium* species consisting of *B. bifidum*, *B. longum*, *Bifidobacterium animalis* subsp. *lactis*, and *B. breve*, which migrate to and integrate cancer cells where they interact with the immune system of the host stimulating an immune signaling pathway called interferon gene stimulation (STING). Essentially, this represents a process that translates into an abundant activation of the immune cells, which allows enhancement of the anti-CD47 therapy by increasing its ability to destroy cancer cells (Shi et al., 2020).

Bacterial therapy protocols are being developed in cancer treatment, based on previous success of studies describing cancer patients entering remission after a bacterial treatment (Enck, 1991). Bacterial therapies are based upon the ability of the microbial cell to selectively interact with and kill cancer cells *in situ* and stimulate a strong anti-cancer immune response (Forbes et al., 2018). Preclinical studies have shown that these therapies retard tumor growth and increase survival (Dang et al., 2001; Ganai et al., 2009). A prime example of a cancer therapy protocol is based on attenuated microbial cells in the treatment of superficial bladder cancer with the *Bacillus* Calmette-Guerin (BCG) vaccine. This therapy likely stimulates the non-specific immune responses against the tumor and represents the only anti-cancer bacterial therapy that is currently considered as an established standard of care (Kamat et al., 2015). Bacterial therapies work mainly by direct oncolysis mediated by the secretion of exotoxins or competition for nutrients (Middlebrook and Dorland, 1984), but, intracellular bacteria can kill the host’s cancer cells by inducing apoptosis or uncontrolled proliferation causing the outbreak of infected cancer cells (Uchugonova et al., 2015). Currently, bacterial therapy is commonly used in cases of metastatic disease for specific targeting of cancerous cells and tissues. For this purpose many active bacteria were designed to colonize only the tumor microenvironment (Kasinskas and Forbes, 2007; Zhang et al., 2014) and to induce cell death specifically in cancer by oncolytic function (St Jean et al., 2014). In addition, designed immune-sensitizing bacteria induce responses to cancer-specific antigens directly (Wood and Paterson, 2014) or indirectly by spreading the epitope (Seavey et al., 2009).

## Conclusion

There is a growing number of studies demonstrating that intestinal microbiota can be linked to positive effects in clinical outcomes of cancer therapy. Modulation of the gut microbiota is one of the ways to counteract cancer, improving responsiveness to anti-cancer therapies, in particular immunotherapies. Bifidobacteria, which are commonly used as probiotics for their health-promoting features, have been shown to improve tumor control to the same degree as immune checkpoint blockade therapy, with combination treatment nearly abolishing tumor outgrowth. However, despite their well-established role in stimulating human health, the precise mechanisms by which bifidobacteria solicit beneficial effects in fighting cancer are far from being fully understood. The importance of this emerging beneficial role in terms of early diagnosis and the effectiveness of therapies is remarkable. Knowing that the composition of the microbiota is predictive for the presence or absence of disease may guide the development of novel, less invasive tests, and may subsequently lead to the development of personalized treatments.

## Author Contributions

GL: writing – original draft preparation. DS, MV, and FT: writing – review, editing, and conceptualization. All authors contributed to the article and approved the submitted version.

## Conflict of Interest

The authors declare that the research was conducted in the absence of any commercial or financial relationships that could be construed as a potential conflict of interest. The reviewer VR declared a shared affiliation, with no collaboration, with one of the authors, DS, to the handling editor at the time of review.

## References

[B1] Abed J. Emgard J. E. Zamir G. Faroja M. Almogy G. Grenov A. (2016). Fap2 mediates *Fusobacterium nucleatum* colorectal adenocarcinoma enrichment by binding to tumor-expressed gal-GalNAc. *Cell Host Microbe* 20 215–225. 10.1016/j.chom.2016.07.006 27512904 PMC5465824

[B2] Advani R. Flinn I. Popplewell L. Forero A. Bartlett N. L. Ghosh N. (2018). CD47 blockade by Hu5F9-G4 and rituximab in non-hodgkin’s lymphoma. *N. Engl. J. Med.* 379 1711–1721. 10.1056/NEJMoa1807315 30380386 PMC8058634

[B3] Alessandri G. Ossiprandi M. C. MacSharry J. van Sinderen D. Ventura M. (2019). Bifidobacterial dialogue with its human host and consequent modulation of the immune system. *Front. Immunol.* 10:2348. 10.3389/fimmu.2019.02348 31632412 PMC6779802

[B4] Alvarez-Martin P. O’Connell Motherway M. Turroni F. Foroni E. Ventura M. van Sinderen D. (2012). A two-component regulatory system controls autoregulated serpin expression in Bifidobacterium breve UCC2003. *Appl. Environ. Microbiol.* 78 7032–7041. 10.1128/AEM.01776-12 22843530 PMC3457475

[B5] Ambalam P. Raman M. Purama R. K. Doble M. (2016). Probiotics, prebiotics and colorectal cancer prevention. *Best Pract. Res. Clin. Gastroenterol.* 30 119–131. 10.1016/j.bpg.2016.02.009 27048903

[B6] Arboleya S. Watkins C. Stanton C. Ross R. P. (2016). Gut bifidobacteria populations in human health and aging. *Front. Microbiol.* 7:1204. 10.3389/fmicb.2016.01204 27594848 PMC4990546

[B7] Arseneau K. O. Cominelli F. (2009). Leukocytapheresis in ulcerative colitis: a possible alternative to biological therapy? *Dig. Liver Dis.* 41 551–552. 10.1016/j.dld.2009.05.014 19540820 PMC3572230

[B8] Azuma T. Yao S. Zhu G. Flies A. S. Flies S. J. Chen L. (2008). B7-H1 is a ubiquitous antiapoptotic receptor on cancer cells. *Blood* 111 3635–3643. 10.1182/blood-2007-11-123141 18223165 PMC2275025

[B9] Bashiardes S. Tuganbaev T. Federici S. Elinav E. (2017). The microbiome in anti-cancer therapy. *Semin. Immunol.* 32 74–81. 10.1016/j.smim.2017.04.001 28431920

[B10] Bertkova I. Hijova E. Chmelarova A. Mojzisova G. Petrasova D. Strojny L. (2010). The effect of probiotic microorganisms and bioactive compounds on chemically induced carcinogenesis in rats. *Neoplasma* 57 422–428. 10.4149/neo_2010_05_422 20568896

[B11] Bezine E. Vignard J. Mirey G. (2014). The cytolethal distending toxin effects on Mammalian cells: a DNA damage perspective. *Cells* 3 592–615. 10.3390/cells3020592 24921185 PMC4092857

[B12] Borghaei H. Paz-Ares L. Horn L. Spigel D. R. Steins M. Ready N. E. (2015). Nivolumab versus docetaxel in advanced nonsquamous non-small-cell lung cancer. *N. Engl. J. Med.* 373 1627–1639. 10.1056/NEJMoa1507643 26412456 PMC5705936

[B13] Brauner A. Brandt L. Frisan T. Thelestam M. Ekbom A. (2010). Is there a risk of cancer development after Campylobacter infection? *Scand. J. Gastroenterol.* 45 893–897. 10.3109/00365521003734133 20334473

[B14] Brennan C. A. Garrett W. S. (2016). Gut microbiota, inflammation, and colorectal cancer. *Annu. Rev. Microbiol.* 70 395–411. 10.1146/annurev-micro-102215-095513 27607555 PMC5541233

[B15] Brennan C. A. Garrett W. S. (2019). *Fusobacterium nucleatum* – symbiont, opportunist and oncobacterium. *Nat. Rev. Microbiol.* 17 156–166. 10.1038/s41579-018-0129-6 30546113 PMC6589823

[B16] Brestoff J. R. Artis D. (2013). Commensal bacteria at the interface of host metabolism and the immune system. *Nat. Immunol.* 14 676–684. 10.1038/ni.2640 23778795 PMC4013146

[B17] Bronte V. Mocellin S. (2009). Suppressive influences in the immune response to cancer. *J. Immunother.* 32 1–11. 10.1097/CJI.0b013e3181837276 19307988

[B18] Buc E. Dubois D. Sauvanet P. Raisch J. Delmas J. Darfeuille-Michaud A. (2013). High prevalence of mucosa-associated E coli producing cyclomodulin and genotoxin in colon cancer. *PLoS ONE* 8:e56964. 10.1371/journal.pone.0056964 23457644 PMC3572998

[B19] Bullman S. Pedamallu C. S. Sicinska E. Clancy T. E. Zhang X. Cai D. (2017). Analysis of *Fusobacterium* persistence and antibiotic response in colorectal cancer. *Science* 358 1443–1448. 10.1126/science.aal5240 29170280 PMC5823247

[B20] Bunesova V. Lacroix C. Schwab C. (2018). Mucin cross-feeding of infant *Bifidobacteria* and *Eubacterium hallii*. *Microb. Ecol.* 75 228–238. 10.1007/s00248-017-1037-4 28721502

[B21] Carbone D. P. Reck M. Paz-Ares L. Creelan B. Horn L. Steins M. (2017). First-line nivolumab in stage IV or recurrent non-small-cell lung cancer. *N. Engl. J. Med.* 376 2415–2426. 10.1056/NEJMoa1613493 28636851 PMC6487310

[B22] Chan A. T. Arber N. Burn J. Chia W. K. Elwood P. Hull M. A. (2012). Aspirin in the chemoprevention of colorectal neoplasia: an overview. *Cancer Prev. Res. (Phila)* 5 164–178. 10.1158/1940-6207.CAPR-11-0391 22084361 PMC3273592

[B23] Chaput N. Lepage P. Coutzac C. Soularue E. Le Roux K. Monot C. (2017). Baseline gut microbiota predicts clinical response and colitis in metastatic melanoma patients treated with ipilimumab. *Ann. Oncol.* 28 1368–1379. 10.1093/annonc/mdx108 28368458

[B24] Chen D. Wu J. Jin D. Wang B. Cao H. (2019). Fecal microbiota transplantation in cancer management: current status and perspectives. *Int. J. Cancer* 145 2021–2031. 10.1002/ijc.32003 30458058 PMC6767494

[B25] Claesson M. J. Cusack O’Sullivan S. O. Greene-Diniz R. de Weerd H. Flannery E. Marchesi J. R. (2011). Composition, variability, and temporal stability of the intestinal microbiota of the elderly. *Proc. Natl. Acad. Sci. U.S.A.* 108(Suppl. 1), 4586–4591. 10.1073/pnas.1000097107 20571116 PMC3063589

[B26] Clemente J. C. Ursell L. K. Parfrey L. W. Knight R. (2012). The impact of the gut microbiota on human health: an integrative view. *Cell* 148 1258–1270. 10.1016/j.cell.2012.01.035 22424233 PMC5050011

[B27] Collado M. C. Derrien M. Isolauri E. de Vos W. M. Salminen S. (2007). Intestinal integrity and Akkermansia muciniphila, a mucin-degrading member of the intestinal microbiota present in infants, adults, and the elderly. *Appl. Environ. Microbiol.* 73 7767–7770. 10.1128/AEM.01477-07 17933936 PMC2168041

[B28] Cronin M. Akin A. R. Collins S. A. Meganck J. Kim J. B. Baban C. K. (2012). High resolution in vivo bioluminescent imaging for the study of bacterial tumour targeting. *PLoS ONE* 7:e30940. 10.1371/journal.pone.0030940 22295120 PMC3266281

[B29] Cuevas-Ramos G. Petit C. R. Marcq I. Boury M. Oswald E. Nougayrede J. P. (2010). *Escherichia coli* induces DNA damage in vivo and triggers genomic instability in mammalian cells. *Proc. Natl. Acad. Sci. U.S.A.* 107 11537–11542. 10.1073/pnas.1001261107 20534522 PMC2895108

[B30] Dalmasso G. Cougnoux A. Delmas J. Darfeuille-Michaud A. Bonnet R. (2014). The bacterial genotoxin colibactin promotes colon tumor growth by modifying the tumor microenvironment. *Gut Microbes* 5 675–680. 10.4161/19490976.2014.969989 25483338 PMC4615906

[B31] Dang L. H. Bettegowda C. Huso D. L. Kinzler K. W. Vogelstein B. (2001). Combination bacteriolytic therapy for the treatment of experimental tumors. *Proc. Natl. Acad. Sci. U.S.A.* 98 15155–15160. 10.1073/pnas.251543698 11724950 PMC64999

[B32] Dapito D. H. Mencin A. Gwak G. Y. Pradere J. P. Jang M. K. Mederacke I. (2012). Promotion of hepatocellular carcinoma by the intestinal microbiota and TLR4. *Cancer Cell* 21 504–516. 10.1016/j.ccr.2012.02.007 22516259 PMC3332000

[B33] Dhaliwal A. Vlachostergios P. J. Oikonomou K. G. Moshenyat Y. (2015). Fecal DNA testing for colorectal cancer screening: molecular targets and perspectives. *World J. Gastrointest. Oncol.* 7 178–183. 10.4251/wjgo.v7.i10.178 26483873 PMC4606173

[B34] Diggs D. L. Huderson A. C. Harris K. L. Myers J. N. Banks L. D. Rekhadevi P. V. (2011). Polycyclic aromatic hydrocarbons and digestive tract cancers: a perspective. *J. Environ. Sci. Health C Environ. Carcinog Ecotoxicol. Rev.* 29 324–357. 10.1080/10590501.2011.629974 22107166 PMC3247201

[B35] Eckburg P. B. Bik E. M. Bernstein C. N. Purdom E. Dethlefsen L. Sargent M. (2005). Diversity of the human intestinal microbial flora. *Science* 308 1635–1638. 10.1126/science.1110591 15831718 PMC1395357

[B36] Ejby M. Fredslund F. Vujicic-Zagar A. Svensson B. Slotboom D. J. Abou M. (2013). Hachem: structural basis for arabinoxylo-oligosaccharide capture by the probiotic Bifidobacterium animalis subsp. *lactis Bl-*04. *Mol. Microbiol.* 90 1100–1112. 10.1111/mmi.12419 24279727

[B37] Elkrief A. Derosa L. Zitvogel L. Kroemer G. Routy B. (2019). The intimate relationship between gut microbiota and cancer immunotherapy. *Gut Microbes* 10 424–428. 10.1080/19490976.2018.1527167 30339501 PMC6546322

[B38] Enck R. E. (1991). Understanding tolerance, physical dependence and addiction in the use of opioid analgesics. *Am. J. Hosp. Palliat Care* 8 9–11. 10.1177/104990919100800102 1888599

[B39] Eribe E. R. K. Paster B. J. Caugant D. A. Dewhirst F. E. Stromberg V. K. Lacy G. H. (2004). Genetic diversity of Leptotrichia and description of *Leptotrichia goodfellowii* sp. *nov*. *Leptotrichia hofstadii* sp. nov., *Leptotrichia shahii* sp. nov. and *Leptotrichia wadei* sp. nov. *Int. J. Syst. Evol. Microbiol.* 54(Pt 2), 583–592. 10.1099/ijs.0.02819-0 15023979

[B40] Fanning S. Hall L. J. Cronin M. Zomer A. MacSharry J. Goulding D. (2012a). Bifidobacterial surface-exopolysaccharide facilitates commensal-host interaction through immune modulation and pathogen protection. *Proc. Natl. Acad. Sci. U.S.A.* 109 2108–2113. 10.1073/pnas.1115621109 22308390 PMC3277520

[B41] Fanning S. Hall L. J. van Sinderen D. (2012b). Bifidobacterium breve UCC2003 surface exopolysaccharide production is a beneficial trait mediating commensal-host interaction through immune modulation and pathogen protection. *Gut Microbes* 3 420–425. 10.4161/gmic.20630 22713271

[B42] Ferrario C. Milani C. Mancabelli L. Lugli G. A. Duranti S. Mangifesta M. (2016). Modulation of the eps-ome transcription of bifidobacteria through simulation of human intestinal environment. *FEMS Microbiol Ecol* 92:fiw056. 10.1093/femsec/fiw056 26960391

[B43] Fessler J. Matson V. Gajewski T. F. (2019). Exploring the emerging role of the microbiome in cancer immunotherapy. *J Immunother. Cancer* 7:108. 10.1186/s40425-019-0574-4 30995949 PMC6471869

[B44] Finn O. J. (2012). Immuno-oncology: understanding the function and dysfunction of the immune system in cancer. *Ann. Oncol.* 23(Suppl. 8), viii6–viii9. 10.1093/annonc/mds256 22918931 PMC4085883

[B45] Forbes N. S. Coffin R. S. Deng L. Evgin L. Fiering S. Giacalone M. (2018). White paper on microbial anti-cancer therapy and prevention. *J. Immunother. Cancer* 6:78. 10.1186/s40425-018-0381-3 30081947 PMC6091193

[B46] Foroni E. Serafini F. Amidani D. Turroni F. He F. Bottacini F. (2011). Genetic analysis and morphological identification of pilus-like structures in members of the genus Bifidobacterium. *Microb. Cell Fact* 10(Suppl. 1), S16. 10.1186/1475-2859-10-S1-S16 21995649 PMC3231923

[B47] Fox J. G. Wang T. C. (2007). Inflammation, atrophy, and gastric cancer. *J. Clin. Invest.* 117 60–69. 10.1172/JCI30111 17200707 PMC1716216

[B48] Francisco L. M. Sage P. T. Sharpe A. H. (2010). The PD-1 pathway in tolerance and autoimmunity. *Immunol. Rev.* 236 219–242. 10.1111/j.1600-065X.2010.00923.x 20636820 PMC2919275

[B49] Freeman G. J. Long A. J. Iwai Y. Bourque K. Chernova T. Nishimura H. (2000). Engagement of the PD-1 immunoinhibitory receptor by a novel B7 family member leads to negative regulation of lymphocyte activation. *J. Exp. Med.* 192 1027–1034. 10.1084/jem.192.7.1027 11015443 PMC2193311

[B50] Fuentes S. de Vos W. M. (2016). How to manipulate the microbiota: fecal microbiota transplantation. *Adv. Exp. Med. Biol.* 902 143–153. 10.1007/978-3-319-31248-4_10 27161356

[B51] Fujimura K. E. Slusher N. A. Cabana M. D. Lynch S. V. (2010). Role of the gut microbiota in defining human health. *Expert Rev. Anti Infect. Ther.* 8 435–454. 10.1586/eri.10.14 20377338 PMC2881665

[B52] Fung K. Y. Cosgrove L. Lockett T. Head R. Topping D. L. (2012). A review of the potential mechanisms for the lowering of colorectal oncogenesis by butyrate. *Br. J. Nutr.* 108 820–831. 10.1017/S0007114512001948 22676885

[B53] Ganai S. Arenas R. B. Forbes N. S. (2009). Tumour-targeted delivery of TRAIL using *Salmonella* typhimurium enhances breast cancer survival in mice. *Br. J. Cancer* 101 1683–1691. 10.1038/sj.bjc.6605403 19861961 PMC2778534

[B54] Garrett W. S. (2015). Cancer and the microbiota. *Science* 348 80–86. 10.1126/science.aaa4972 25838377 PMC5535753

[B55] Ge Z. Feng Y. Ge L. Parry N. Muthupalani S. Fox J. G. (2017). *Helicobacter* hepaticus cytolethal distending toxin promotes intestinal carcinogenesis in 129Rag2-deficient mice. *Cell Microbiol.* 19. 10.1111/cmi.12728 28111881 PMC5469717

[B56] Ge Z. Rogers A. B. Feng Y. Lee A. Xu S. Taylor N. S. (2007). Bacterial cytolethal distending toxin promotes the development of dysplasia in a model of microbially induced hepatocarcinogenesis. *Cell Microbiol.* 9 2070–2080. 10.1111/j.1462-5822.2007.00939.x 17441986

[B57] Geller L. T. Barzily-Rokni M. Danino T. Jonas O. H. Shental N. Nejman D. (2017). Potential role of intratumor bacteria in mediating tumor resistance to the chemotherapeutic drug gemcitabine. *Science* 357 1156–1160. 10.1126/science.aah5043 28912244 PMC5727343

[B58] Gevers D. Kugathasan S. Denson L. A. Van Treuren W. Ren B. (2014). The treatment-naive microbiome in new-onset Crohn’s disease. *Cell Host Microbe* 15 382–392. 10.1016/j.chom.2014.02.005 24629344 PMC4059512

[B59] Gibson G. R. Hutkins R. Sanders M. E. Prescott S. L. Reimer R. A. Salminen S. J. (2017). Expert consensus document: the international scientific association for probiotics and prebiotics (ISAPP) consensus statement on the definition and scope of prebiotics. *Nat. Rev. Gastroenterol. Hepatol.* 14 491–502. 10.1038/nrgastro.2017.75 28611480

[B60] Gobel C. Breitenbuecher F. Kalkavan H. Hahnel P. S. Kasper S. Hoffarth S. (2014). Functional expression cloning identifies COX-2 as a suppressor of antigen-specific cancer immunity. *Cell Death Dis.* 5:e1568. 10.1038/cddis.2014.531 25501829 PMC4649842

[B61] Gopalakrishnan V. Helmink B. A. Spencer C. N. Reuben A. Wargo J. A. (2018a). The influence of the gut microbiome on cancer, immunity, and cancer immunotherapy. *Cancer Cell* 33 570–580. 10.1016/j.ccell.2018.03.015 29634945 PMC6529202

[B62] Gopalakrishnan V. Spencer C. N. Nezi L. Reuben A. Andrews M. C. Karpinets T. V. (2018b). Gut microbiome modulates response to anti-PD-1 immunotherapy in melanoma patients. *Science* 359 97–103. 10.1126/science.aan4236 29097493 PMC5827966

[B63] Grillot-Courvalin C. Goussard S. Huetz F. Ojcius D. M. Courvalin P. (1998). Functional gene transfer from intracellular bacteria to mammalian cells. *Nat. Biotechnol.* 16 862–866. 10.1038/nbt0998-862 9743121

[B64] Guerra L. Cortes-Bratti X. Guidi R. Frisan T. (2011). The biology of the cytolethal distending toxins. *Toxins (Basel)* 3 172–190. 10.3390/toxins3030172 22069704 PMC3202825

[B65] Guglielmetti S. Tamagnini I. Mora D. Minuzzo M. Scarafoni A. Arioli S. (2008). Implication of an outer surface lipoprotein in adhesion of Bifidobacterium bifidum to Caco-2 cells. *Appl. Environ. Microbiol.* 74 4695–4702. 10.1128/AEM.00124-08 18539800 PMC2519326

[B66] Guglielmetti S. Zanoni I. Balzaretti S. Miriani M. Taverniti V. De Noni I. (2014). Murein lytic enzyme TgaA of Bifidobacterium bifidum MIMBb75 modulates dendritic cell maturation through its cysteine- and histidine-dependent amidohydrolase/peptidase (CHAP) amidase domain. *Appl. Environ. Microbiol.* 80 5170–5177. 10.1128/AEM.00761-14 24814791 PMC4136110

[B67] Guinane C. M. Cotter P. D. (2013). Role of the gut microbiota in health and chronic gastrointestinal disease: understanding a hidden metabolic organ. *Therap. Adv. Gastroenterol.* 6 295–308. 10.1177/1756283X13482996 23814609 PMC3667473

[B68] Gur C. Ibrahim Y. Isaacson B. Yamin R. Abed J. Gamliel M. (2015). Binding of the Fap2 protein of *Fusobacterium nucleatum* to human inhibitory receptor TIGIT protects tumors from immune cell attack. *Immunity* 42 344–355. 10.1016/j.immuni.2015.01.010 25680274 PMC4361732

[B69] Gursoy U. K. Pollanen M. Kononen E. Uitto V. J. (2010). Biofilm formation enhances the oxygen tolerance and invasiveness of *Fusobacterium nucleatum* in an oral mucosa culture model. *J. Periodontol.* 81 1084–1091. 10.1902/jop.2010.090664 20350156

[B70] Hambly R. J. Rumney C. J. Fletcher J. M. Rijken P. J. Rowland I. R. (1997). Effects of high- and low-risk diets on gut microflora-associated biomarkers of colon cancer in human flora-associated rats. *Nutr. Cancer* 27 250–255. 10.1080/01635589709514534 9101554

[B71] Hamer H. M. Jonkers D. Venema K. Vanhoutvin S. Troost F. J. Brummer R. J. (2008). Review article: the role of butyrate on colonic function. *Aliment. Pharmacol. Ther.* 27 104–119. 10.1111/j.1365-2036.2007.03562.x 17973645

[B72] Han X. Y. Weinberg J. S. Prabhu S. S. Hassenbusch S. J. Fuller G. N. Tarrand J. J. (2003). Fusobacterial brain abscess: a review of five cases and an analysis of possible pathogenesis. *J. Neurosurg.* 99 693–700. 10.3171/jns.2003.99.4.0693 14567605

[B73] Han Y. W. Shi W. Huang G. T. Park N. H. Kuramitsu H. (2000). Interactions between periodontal bacteria and human oral epithelial cells: *Fusobacterium nucleatum* adheres to and invades epithelial cells. *Infect. Immun.* 68 3140–3146. 10.1128/iai.68.6.3140-3146.2000 10816455 PMC97547

[B74] Hanahan D. Weinberg R. A. (2011). Hallmarks of cancer: the next generation. *Cell* 144 646–674. 10.1016/j.cell.2011.02.013 21376230

[B75] He Z. Gharaibeh R. Z. Newsome R. C. Pope J. L. Dougherty M. W. Tomkovich S. (2019). Campylobacter jejuni promotes colorectal tumorigenesis through the action of cytolethal distending toxin. *Gut* 68 289–300. 10.1136/gutjnl-2018-317200 30377189 PMC6352414

[B76] Helmink B. A. Khan M. A. W. Hermann A. Gopalakrishnan V. Wargo J. A. (2019). The microbiome, cancer, and cancer therapy. *Nat. Med.* 25 377–388. 10.1038/s41591-019-0377-7 30842679

[B77] Helmy K. Y. Patel S. A. Nahas G. R. Rameshwar P. (2013). Cancer immunotherapy: accomplishments to date and future promise. *Ther. Deliv.* 4 1307–1320. 10.4155/tde.13.88 24116914

[B78] Hidalgo-Cantabrana C. Delgado S. Ruiz L. Ruas-Madiedo P. Sanchez B. Margolles A. (2017). Bifidobacteria and their health-promoting effects. *Microbiol. Spectr.* 5. 10.1128/microbiolspec.BAD-0010-2016 28643627 PMC11687494

[B79] Hidalgo-Cantabrana C. Nikolic M. Lopez P. Suarez A. Miljkovic M. Kojic M. (2014). Exopolysaccharide-producing *Bifidobacterium animalis* subsp. lactis strains and their polymers elicit different responses on immune cells from blood and gut associated lymphoid tissue. *Anaerobe* 26 24–30. 10.1016/j.anaerobe.2014.01.003 24445155

[B80] Hope M. E. Hold G. L. Kain R. El-Omar E. M. (2005). Sporadic colorectal cancer–role of the commensal microbiota. *FEMS Microbiol. Lett.* 244 1–7. 10.1016/j.femsle.2005.01.029 15727814

[B81] Hughes K. R. Harnisch L. C. Alcon-Giner C. Mitra S. Wright C. J. Ketskemety J. (2017). Bifidobacterium breve reduces apoptotic epithelial cell shedding in an exopolysaccharide and MyD88-dependent manner. *Open Biol.* 7:160155. 10.1098/rsob.160155 28123052 PMC5303268

[B82] Illiano P. Brambilla R. Parolini C. (2020). The mutual interplay of gut microbiota, diet and human disease. *FEBS J.* 287 833–855. 10.1111/febs.15217 31955527

[B83] Jemal A. Bray F. Center M. M. Ferlay J. Ward E. Forman D. (2011). Global cancer statistics. *CA Cancer J. Clin.* 61 69–90. 10.3322/caac.20107 21296855

[B84] Kai A. Cooke F. Antoun N. Siddharthan C. Sule O. (2008). A rare presentation of ventriculitis and brain abscess caused by *Fusobacterium nucleatum*. *J. Med. Microbiol.* 57(Pt 5), 668–671. 10.1099/jmm.0.47710-0 18436604

[B85] Kainulainen V. Reunanen J. Hiippala K. Guglielmetti S. Vesterlund S. Palva A. (2013). BopA does not have a major role in the adhesion of Bifidobacterium bifidum to intestinal epithelial cells, extracellular matrix proteins, and mucus. *Appl. Environ. Microbiol.* 79 6989–6997. 10.1128/AEM.01993-13 24014530 PMC3811531

[B86] Kalos M. Levine B. L. Porter D. L. Katz S. Grupp S. A. Bagg A. (2011). T cells with chimeric antigen receptors have potent antitumor effects and can establish memory in patients with advanced leukemia. *Sci. Transl. Med.* 3:95ra73. 10.1126/scitranslmed.3002842 21832238 PMC3393096

[B87] Kamat A. M. Flaig T. W. Grossman H. B. Konety B. Lamm D. O’Donnell M. A. (2015). Expert consensus document: consensus statement on best practice management regarding the use of intravesical immunotherapy with BCG for bladder cancer. *Nat. Rev. Urol.* 12 225–235. 10.1038/nrurol.2015.58 25800393

[B88] Kapatral V. Anderson I. Ivanova N. Reznik G. Los T. Lykidis A. (2002). Genome sequence and analysis of the oral bacterium *Fusobacterium nucleatum* strain ATCC 25586. *J. Bacteriol.* 184 2005–2018. 10.1128/jb.184.7.2005-2018.2002 11889109 PMC134920

[B89] Kaplan C. W. Lux R. Haake S. K. Shi W. (2009). The *Fusobacterium nucleatum* outer membrane protein RadD is an arginine-inhibitable adhesin required for inter-species adherence and the structured architecture of multispecies biofilm. *Mol. Microbiol.* 71 35–47. 10.1111/j.1365-2958.2008.06503.x 19007407 PMC2741168

[B90] Kasinskas R. W. Forbes N. S. (2007). *Salmonella* typhimurium lacking ribose chemoreceptors localize in tumor quiescence and induce apoptosis. *Cancer Res.* 67 3201–3209. 10.1158/0008-5472.CAN-06-2618 17409428

[B91] Kinross J. M. Darzi A. W. Nicholson J. K. (2011). Gut microbiome-host interactions in health and disease. *Genome Med.* 3:14. 10.1186/gm228 21392406 PMC3092099

[B92] Kline K. A. Dodson K. W. Caparon M. G. Hultgren S. J. (2010). A tale of two pili: assembly and function of pili in bacteria. *Trends Microbiol.* 18 224–232. 10.1016/j.tim.2010.03.002 20378353 PMC3674877

[B93] Knasmuller S. Steinkellner H. Hirschl A. M. Rabot S. Nobis E. C. Kassie F. (2001). Impact of bacteria in dairy products and of the intestinal microflora on the genotoxic and carcinogenic effects of heterocyclic aromatic amines. *Mutat. Res.* 480–481 129–138. 10.1016/s0027-5107(01)00176-2 11506806

[B94] Kochenderfer J. N. Wilson W. H. Janik J. E. Dudley M. E. Stetler-Stevenson M. Feldman S. A. (2010). Eradication of B-lineage cells and regression of lymphoma in a patient treated with autologous T cells genetically engineered to recognize CD19. *Blood* 116 4099–4102. 10.1182/blood-2010-04-281931 20668228 PMC2993617

[B95] Kolenbrander P. E. Palmer R. J. Jr. Periasamy S. Jakubovics N. S. (2010). Oral multispecies biofilm development and the key role of cell-cell distance. *Nat. Rev. Microbiol.* 8 471–480. 10.1038/nrmicro2381 20514044

[B96] Komiya Y. Shimomura Y. Higurashi T. Sugi Y. Arimoto J. Umezawa S. (2019). Patients with colorectal cancer have identical strains of *Fusobacterium nucleatum* in their colorectal cancer and oral cavity. *Gut* 68 1335–1337. 10.1136/gutjnl-2018-316661 29934439 PMC6582823

[B97] Kostic A. D. Chun E. Robertson L. Glickman J. N. Gallini C. A. Michaud M. (2013). *Fusobacterium nucleatum* potentiates intestinal tumorigenesis and modulates the tumor-immune microenvironment. *Cell Host Microbe* 14 207–215. 10.1016/j.chom.2013.07.007 23954159 PMC3772512

[B98] Kostic A. D. Gevers D. Pedamallu C. S. Michaud M. Duke F. Earl A. M. (2012). Genomic analysis identifies association of *Fusobacterium* with colorectal carcinoma. *Genome Res.* 22 292–298. 10.1101/gr.126573.111 22009990 PMC3266036

[B99] Krisanaprakornkit S. Kimball J. R. Weinberg A. Darveau R. P. Bainbridge B. W. Dale B. A. (2000). Inducible expression of human beta-defensin 2 by *Fusobacterium nucleatum* in oral epithelial cells: multiple signaling pathways and role of commensal bacteria in innate immunity and the epithelial barrier. *Infect. Immun.* 68 2907–2915. 10.1128/iai.68.5.2907-2915.2000 10768988 PMC97503

[B100] Krummel M. F. Allison J. P. (1995). CD28 and CTLA-4 have opposing effects on the response of T cells to stimulation. *J. Exp. Med.* 182 459–465. 10.1084/jem.182.2.459 7543139 PMC2192127

[B101] Latchman Y. Wood C. R. Chernova T. Chaudhary D. Borde M. Chernova I. (2001). PD-L2 is a second ligand for PD-1 and inhibits T cell activation. *Nat. Immunol.* 2 261–268. 10.1038/85330 11224527

[B102] Lauder A. P. Roche A. M. Sherrill-Mix S. Bailey A. Laughlin A. L. Bittinger K. (2016). Comparison of placenta samples with contamination controls does not provide evidence for a distinct placenta microbiota. *Microbiome* 4:29. 10.1186/s40168-016-0172-3 27338728 PMC4917942

[B103] Laursen M. F. Andersen L. B. Michaelsen K. F. Molgaard C. Trolle E. Bahl M. I. (2016). Infant gut microbiota development is driven by transition to family foods independent of maternal obesity. *mSphere* 1:e69-15. 10.1128/mSphere.00069-15 27303699 PMC4863607

[B104] Le D. T. Uram J. N. Wang H. Bartlett B. R. Kemberling H. Eyring A. D. (2015). PD-1 blockade in tumors with mismatch-repair deficiency. *N. Engl. J. Med.* 372 2509–2520. 10.1056/NEJMoa1500596 26028255 PMC4481136

[B105] Linares D. M. Gomez C. Renes E. Fresno J. M. Tornadijo M. E. Ross R. P. (2017). Lactic acid bacteria and bifidobacteria with potential to design natural biofunctional health-promoting dairy foods. *Front. Microbiol.* 8:846. 10.3389/fmicb.2017.00846 28572792 PMC5435742

[B106] Liu P. F. Shi W. Zhu W. Smith J. W. Hsieh S. L. Gallo R. L. (2010). Vaccination targeting surface FomA of *Fusobacterium nucleatum* against bacterial co-aggregation: implication for treatment of periodontal infection and halitosis. *Vaccine* 28 3496–3505. 10.1016/j.vaccine.2010.02.047 20189489 PMC2855893

[B107] Llopis M. Antolin M. Carol M. Borruel N. Casellas F. Martinez C. (2009). Lactobacillus casei downregulates commensals’ inflammatory signals in Crohn’s disease mucosa. *Inflamm. Bowel Dis.* 15 275–283. 10.1002/ibd.20736 18839424

[B108] Lofgren J. L. Whary M. T. Ge Z. Muthupalani S. Taylor N. S. Mobley M. (2011). Lack of commensal flora in *Helicobacter* pylori-infected INS-GAS mice reduces gastritis and delays intraepithelial neoplasia. *Gastroenterology* 140 210–220. 10.1053/j.gastro.2010.09.048 20950613 PMC3006487

[B109] Ma X. Aoki T. Tsuruyama T. Narumiya S. (2015). Definition of prostaglandin E2-EP2 signals in the colon tumor microenvironment that amplify inflammation and tumor growth. *Cancer Res.* 75 2822–2832. 10.1158/0008-5472.CAN-15-0125 26018088

[B110] Mancabelli L. Milani C. Lugli G. A. Fontana F. Turroni F. van Sinderen D. (2020). The impact of primer design on amplicon-based metagenomic profiling accuracy: detailed insights into bifidobacterial community structure. *Microorganisms* 8:131. 10.3390/microorganisms8010131 31963501 PMC7023036

[B111] Manson A. McGuire Cochrane K. Griggs A. D. Haas B. J. Abeel T. Zeng Q. (2014). Evolution of invasion in a diverse set of *Fusobacterium* species. *mBio* 5:e01864. 10.1128/mBio.01864-14 25370491 PMC4222103

[B112] Marteau P. R. de Vrese M. Cellier C. J. Schrezenmeir J. (2001). Protection from gastrointestinal diseases with the use of probiotics. *Am. J. Clin. Nutr.* 73(2 Suppl.), 430S–436S. 10.1093/ajcn/73.2.430s 11157353

[B113] Martin R. Miquel S. Benevides L. Bridonneau C. Robert V. Hudault S. (2017). Functional characterization of novel *Faecalibacterium prausnitzii* strains isolated from healthy volunteers: a step forward in the use of prausnitzii, F., as a next-generation probiotic. *Front. Microbiol.* 8:1226. 10.3389/fmicb.2017.01226 28713353 PMC5492426

[B114] Matson V. Fessler J. Bao R. Chongsuwat T. Zha Y. Alegre M. L. (2018). The commensal microbiome is associated with anti-PD-1 efficacy in metastatic melanoma patients. *Science* 359 104–108. 10.1126/science.aao3290 29302014 PMC6707353

[B115] Maude S. L. Laetsch T. W. Buechner J. Rives S. Boyer M. Bittencourt H. (2018). Tisagenlecleucel in children and young adults with B-cell lymphoblastic leukemia. *N. Engl. J. Med.* 378 439–448. 10.1056/NEJMoa1709866 29385370 PMC5996391

[B116] McCarthy E. F. (2006). The toxins of william coley, B., and the treatment of bone and soft-tissue sarcomas. *Iowa Orthop. J.* 26 154–158. 16789469 PMC1888599

[B117] McCarville J. L. Dong J. Caminero A. Bermudez-Brito M. Jury J. Murray J. A. (2017). A commensal *Bifidobacterium longum* strain prevents gluten-related immunopathology in mice through expression of a serine protease inhibitor. *Appl. Environ. Microbiol.* 83:e1323-17. 10.1128/AEM.01323-17 28778891 PMC5601352

[B118] McCoy A. N. Araujo-Perez F. Azcarate-Peril A. Yeh J. J. Sandler R. S. Keku T. O. (2013). Fusobacterium is associated with colorectal adenomas. *PLoS ONE* 8:e53653. 10.1371/journal.pone.0053653 23335968 PMC3546075

[B119] Middlebrook J. L. Dorland R. B. (1984). Bacterial toxins: cellular mechanisms of action. *Microbiol. Rev.* 48 199–221. 6436655 10.1128/mr.48.3.199-221.1984 PMC373009

[B120] Milani C. Duranti S. Bottacini F. Casey E. Turroni F. Mahony J. (2017a). The first microbial colonizers of the human gut: composition, activities, and health implications of the infant gut microbiota. *Microbiol. Mol. Biol. Rev.* 81:e36-17. 10.1128/MMBR.00036-17 29118049 PMC5706746

[B121] Milani C. Mangifesta M. Mancabelli L. Lugli G. A. Mancino W. Viappiani A. (2017b). The sortase-dependent fimbriome of the genus bifidobacterium: extracellular structures with potential to modulate microbe-host dialogue. *Appl. Environ. Microbiol.* 83:e1295-17. 10.1128/AEM.01295-17 28754709 PMC5601332

[B122] Milani C. Ferrario C. Turroni F. Duranti S. Mangifesta M. van Sinderen D. (2016). The human gut microbiota and its interactive connections to diet. *J. Hum. Nutr. Diet.* 29 539–546. 10.1111/jhn.12371 27161433

[B123] Milani C. Lugli G. A. Duranti S. Turroni F. Mancabelli L. Ferrario C. (2015). Bifidobacteria exhibit social behavior through carbohydrate resource sharing in the gut. *Sci. Rep.* 5:15782. 10.1038/srep15782 26506949 PMC4623478

[B124] Mima K. Nishihara R. Qian Z. R. Cao Y. Sukawa Y. Nowak J. A. (2016). *Fusobacterium nucleatum* in colorectal carcinoma tissue and patient prognosis. *Gut* 65 1973–1980. 10.1136/gutjnl-2015-310101 26311717 PMC4769120

[B125] Molinero N. Ruiz L. Sanchez B. Margolles A. Delgado S. (2019). Intestinal bacteria interplay with bile and cholesterol metabolism: implications on host physiology. *Front. Physiol.* 10:185. 10.3389/fphys.2019.00185 30923502 PMC6426790

[B126] Morgillo F. Dallio M. Della Corte C. M. Gravina A. G. Viscardi G. Loguercio C. (2018). Carcinogenesis as a result of multiple inflammatory and oxidative hits: a comprehensive review from tumor microenvironment to gut microbiota. *Neoplasia* 20 721–733. 10.1016/j.neo.2018.05.002 29859426 PMC6014569

[B127] Motzer R. J. Escudier B. McDermott D. F. George S. Hammers H. J. Srinivas S. (2015). Nivolumab versus everolimus in advanced renal-cell carcinoma. *N. Engl. J. Med.* 373 1803–1813. 10.1056/NEJMoa1510665 26406148 PMC5719487

[B128] Nuriel-Ohayon M. Neuman H. Koren O. (2016). Microbial changes during pregnancy, birth, and infancy. *Front. Microbiol.* 7:1031. 10.3389/fmicb.2016.01031 27471494 PMC4943946

[B129] Nyfors S. Kononen E. Syrjanen R. Komulainen E. Jousimies-Somer H. (2003). Emergence of penicillin resistance among *Fusobacterium nucleatum* populations of commensal oral flora during early childhood. *J. Antimicrob. Chemother.* 51 107–112. 10.1093/jac/dkg022 12493794

[B130] O’Callaghan A. van Sinderen D. (2016). Bifidobacteria and their role as members of the human gut microbiota. *Front. Microbiol.* 7:925. 10.3389/fmicb.2016.00925 27379055 PMC4908950

[B131] O’Connell Motherway M. Houston A. O’Callaghan G. Reunanen J. O’Brien F. O. Driscoll T. (2019). A bifidobacterial pilus-associated protein promotes colonic epithelial proliferation. *Mol. Microbiol.* 111 287–301. 10.1111/mmi.14155 30352131

[B132] O’Connell Motherway M. Zomer A. Leahy S. C. Reunanen J. Bottacini F. Claesson M. J. (2011). Functional genome analysis of *Bifidobacterium breve* UCC2003 reveals type IVb tight adherence (Tad) pili as an essential and conserved host-colonization factor. *Proc. Natl. Acad. Sci. U.S.A.* 108 11217–11222. 10.1073/pnas.1105380108 21690406 PMC3131351

[B133] O’Mahony L. Feeney M. O’Halloran S. Murphy L. Kiely B. Fitzgibbon J. (2001). Probiotic impact on microbial flora, inflammation and tumour development in IL-10 knockout mice. *Aliment. Pharmacol. Ther.* 15 1219–1225. 10.1046/j.1365-2036.2001.01027.x 11472326

[B134] Palmer C. Bik E. M. DiGiulio D. B. Relman D. A. Brown P. O. (2007). Development of the human infant intestinal microbiota. *PLoS Biol.* 5:e177. 10.1371/journal.pbio.0050177 17594176 PMC1896187

[B135] Pardoll D. M. (2012). The blockade of immune checkpoints in cancer immunotherapy. *Nat. Rev. Cancer* 12 252–264. 10.1038/nrc3239 22437870 PMC4856023

[B136] Peng B. J. Cao C. Y. Li W. Zhou Y. J. Zhang Y. Nie Y. Q. (2018). Diagnostic performance of intestinal *Fusobacterium nucleatum* in colorectal cancer: a meta-analysis. *Chin. Med. J. (Engl.)* 131 1349–1356. 10.4103/0366-6999.232814 29786050 PMC5987508

[B137] Petrof E. O. Claud E. C. Gloor G. B. Allen-Vercoe E. (2013). Microbial ecosystems therapeutics: a new paradigm in medicine? *Benef. Microbes* 4 53–65. 10.3920/BM2012.0039 23257018

[B138] Pickard J. M. Zeng M. Y. Caruso R. Nunez G. (2017). Gut microbiota: role in pathogen colonization, immune responses, and inflammatory disease. *Immunol. Rev.* 279 70–89. 10.1111/imr.12567 28856738 PMC5657496

[B139] Pithva S. P. Ambalam P. S. Ramoliya J. M. Dave J. M. Vyas B. R. (2015). Antigenotoxic and antimutagenic activities of probiotic *Lactobacillus rhamnosus* Vc against N-methyl-N’-Nitro-N-nitrosoguanidine. *Nutr. Cancer* 67 1142–1150. 10.1080/01635581.2015.1073751 26312410

[B140] Potempa J. Korzus E. Travis J. (1994). The serpin superfamily of proteinase inhibitors: structure, function, and regulation. *J. Biol. Chem.* 269 15957–15960. 8206889

[B141] Prakash S. Rodes L. Coussa-Charley M. Tomaro-Duchesneau C. (2011). Gut microbiota: next frontier in understanding human health and development of biotherapeutics. *Biologics* 5 71–86. 10.2147/BTT.S19099 21847343 PMC3156250

[B142] Prieto P. A. Yang J. C. Sherry R. M. Hughes M. S. Kammula U. S. White D. E. (2012). CTLA-4 blockade with ipilimumab: long-term follow-up of 177 patients with metastatic melanoma. *Clin. Cancer Res.* 18 2039–2047. 10.1158/1078-0432.CCR-11-1823 22271879 PMC3319861

[B143] Pryde S. E. Duncan S. H. Hold G. L. Stewart C. S. Flint H. J. (2002). The microbiology of butyrate formation in the human colon. *FEMS Microbiol. Lett.* 217 133–139. 10.1111/j.1574-6968.2002.tb11467.x 12480096

[B144] Pungel D. Treveil A. Dalby M. J. Caim S. Colquhoun I. J. Booth C. (2020). *Bifidobacterium breve* UCC2003 exopolysaccharide modulates the early life microbiota by acting as a potential dietary substrate. *Nutrients* 12:948. 10.3390/nu12040948 32235410 PMC7231044

[B145] Putignani L. Del Chierico F. Petrucca A. Vernocchi P. Dallapiccola B. (2014). The human gut microbiota: a dynamic interplay with the host from birth to senescence settled during childhood. *Pediatr. Res.* 76 2–10. 10.1038/pr.2014.49 24732106

[B146] Puzanov I. Diab A. Abdallah K. Bingham C. O. III Brogdon C. Dadu R. (2017). for Immunotherapy of cancer toxicity management working: managing toxicities associated with immune checkpoint inhibitors: consensus recommendations from the society for immunotherapy of cancer (SITC) toxicity management working group. *J. Immunother. Cancer* 5:95. 10.1186/s40425-017-0300-z 29162153 PMC5697162

[B147] Rafter J. (2003). Probiotics and colon cancer. *Best Pract. Res. Clin. Gastroenterol.* 17 849–859. 10.1016/S1521-6918(03)00056-8 14507593

[B148] Rajilic-Stojanovic M. Smidt H. de Vos W. M. (2007). Diversity of the human gastrointestinal tract microbiota revisited. *Environ. Microbiol.* 9 2125–2136. 10.1111/j.1462-2920.2007.01369.x 17686012

[B149] Raman M. Ambalam P. Kondepudi K. K. Pithva S. Kothari C. Patel A. T. (2013). Potential of probiotics, prebiotics and synbiotics for management of colorectal cancer. *Gut. Microbes* 4 181–192. 10.4161/gmic.23919 23511582 PMC3669163

[B150] Rautava S. Luoto R. Salminen S. Isolauri E. (2012). Microbial contact during pregnancy, intestinal colonization and human disease. *Nat. Rev. Gastroenterol. Hepatol.* 9 565–576. 10.1038/nrgastro.2012.144 22890113

[B151] Reig M. Baquero F. Garcia-Campello M. Loza E. (1985). *Leptotrichia buccalis* bacteremia in neutropenic children. *J. Clin. Microbiol.* 22 320–321. 4031045 10.1128/jcm.22.2.320-321.1985 PMC268389

[B152] Riordan T. (2007). Human infection with *Fusobacterium necrophorum* (Necrobacillosis), with a focus on Lemierre’s syndrome. *Clin. Microbiol. Rev.* 20 622–659. 10.1128/CMR.00011-07 17934077 PMC2176048

[B153] Rios-Covian D. Gueimonde M. Duncan S. H. Flint H. J. de los Reyes-Gavilan C. G. (2015). Enhanced butyrate formation by cross-feeding between *Faecalibacterium prausnitzii* and *Bifidobacterium adolescentis*. *FEMS Microbiol. Lett.* 362:fnv176. 10.1093/femsle/fnv176 26420851

[B154] Riviere A. Gagnon M. Weckx S. Roy D. De Vuyst L. (2015). Mutual cross-feeding interactions between *Bifidobacterium longum* subsp. longum *NCC*2705 and *Eubacterium rectale* ATCC 33656 explain the bifidogenic and butyrogenic effects of arabinoxylan oligosaccharides. *Appl. Environ. Microbiol.* 81 7767–7781. 10.1128/AEM.02089-15 26319874 PMC4616955

[B155] Roberfroid M. (2007). Prebiotics: the concept revisited. *J. Nutr.* 137(3 Suppl. 2), 830S–837S. 10.1093/jn/137.3.830S 17311983

[B156] Robertson A. G. Kim J. Al-Ahmadie H. Bellmunt J. Guo G. Cherniack A. D. (2017). Comprehensive molecular characterization of muscle-invasive bladder cancer. *Cell* 171 540–556.e25. 10.1016/j.cell.2017.09.007 28988769 PMC5687509

[B157] Rodriguez J. M. Murphy K. Stanton C. Ross R. P. Kober O. I. Juge N. (2015). The composition of the gut microbiota throughout life, with an emphasis on early life. *Microb. Ecol. Health Dis.* 26:26050. 10.3402/mehd.v26.26050 25651996 PMC4315782

[B158] Rogowski A. Briggs J. A. Mortimer J. C. Tryfona T. Terrapon N. Lowe E. C. (2015). Glycan complexity dictates microbial resource allocation in the large intestine. *Nat. Commun.* 6:7481. 10.1038/ncomms8481 26112186 PMC4491172

[B159] Routy B. Le Chatelier E. Derosa L. Duong C. P. M. Alou M. T. Daillere R. (2018). Gut microbiome influences efficacy of PD-1-based immunotherapy against epithelial tumors. *Science* 359 91–97. 10.1126/science.aan3706 29097494

[B160] Rowland I. Gibson G. Heinken A. Scott K. Swann J. Thiele I. (2018). Gut microbiota functions: metabolism of nutrients and other food components. *Eur. J. Nutr.* 57 1–24. 10.1007/s00394-017-1445-8 28393285 PMC5847071

[B161] Ruas-Madiedo P. Gueimonde M. Arigoni F. de los Reyes-Gavilan C. G. Margolles A. (2009). Bile affects the synthesis of exopolysaccharides by *Bifidobacterium animalis*. *Appl. Environ. Microbiol.* 75 1204–1207. 10.1128/AEM.00908-08 19088310 PMC2643586

[B162] Rubinstein M. R. Wang X. Liu W. Hao Y. Cai G. Han Y. W. (2013). *Fusobacterium nucleatum* promotes colorectal carcinogenesis by modulating E-cadherin/beta-catenin signaling via its FadA adhesin. *Cell Host Microbe* 14 195–206. 10.1016/j.chom.2013.07.012 23954158 PMC3770529

[B163] Ruiz L. Delgado S. Ruas-Madiedo P. Sanchez B. Margolles A. (2017). Bifidobacteria and their molecular communication with the immune system. *Front. Microbiol.* 8:2345. 10.3389/fmicb.2017.02345 29255450 PMC5722804

[B164] Salazar N. Lopez P. Garrido P. Moran J. Cabello E. Gueimonde M. (2014). Immune modulating capability of two exopolysaccharide-producing *Bifidobacterium* strains in a wistar rat model. *Biomed. Res. Int.* 2014:106290. 10.1155/2014/106290 24971309 PMC4058098

[B165] Schachter J. Ribas A. Long G. V. Arance A. Grob J. J. Mortier L. (2017). Pembrolizumab versus ipilimumab for advanced melanoma: final overall survival results of a multicentre, randomised, open-label phase 3 study (KEYNOTE-006). *Lancet* 390 1853–1862. 10.1016/S0140-6736(17)31601-X 28822576

[B166] Scheppach W. (1994). Effects of short chain fatty acids on gut morphology and function. *Gut* 35(1 Suppl.), S35–S38. 10.1136/gut.35.1_suppl.s35 8125387 PMC1378144

[B167] Schwab C. Ruscheweyh H. J. Bunesova V. Pham V. T. Beerenwinkel N. Lacroix C. (2017). Trophic interactions of infant bifidobacteria and *Eubacterium hallii* during L-fucose and fucosyllactose degradation. *Front. Microbiol.* 8:95. 10.3389/fmicb.2017.00095 28194144 PMC5277004

[B168] Schwabe R. F. Jobin C. (2013). The microbiome and cancer. *Nat. Rev. Cancer* 13 800–812. 10.1038/nrc3610 24132111 PMC3986062

[B169] Scott J. R. Zahner D. (2006). Pili with strong attachments: gram-positive bacteria do it differently. *Mol. Microbiol.* 62 320–330. 10.1111/j.1365-2958.2006.05279.x 16978260

[B170] Seavey M. M. Maciag P. C. Al-Rawi N. Sewell D. Paterson Y. (2009). An anti-vascular endothelial growth factor receptor 2/fetal liver kinase-1 Listeria monocytogenes anti-angiogenesis cancer vaccine for the treatment of primary and metastatic Her-2/neu+ breast tumors in a mouse model. *J. Immunol.* 182 5537–5546. 10.4049/jimmunol.0803742 19380802 PMC2850569

[B171] Sermer D. Brentjens R. (2019). CAR T-cell therapy: full speed ahead. *Hematol. Oncol.* 37(Suppl. 1), 95–100. 10.1002/hon.2591 31187533

[B172] Shang F. M. Liu H. L. (2018). *Fusobacterium nucleatum* and colorectal cancer: a review. *World J. Gastrointest. Oncol.* 10 71–81. 10.4251/wjgo.v10.i3.71 29564037 PMC5852398

[B173] Sharma P. Allison J. P. (2015). The future of immune checkpoint therapy. *Science* 348 56–61. 10.1126/science.aaa8172 25838373

[B174] Shepard H. M. Phillips G. L. Thanos C. D. Feldmann M. (2017). Developments in therapy with monoclonal antibodies and related proteins. *Clin. Med. (Lond.)* 17 220–232. 10.7861/clinmedicine.17-3-220 28572223 PMC6297577

[B175] Shi Y. Zheng W. Yang K. Harris K. G. Ni K. Xue L. (2020). Intratumoral accumulation of gut microbiota facilitates CD47-based immunotherapy via STING signaling. *J. Exp. Med.* 217:e20192282. 10.1084/jem.20192282 32142585 PMC7201921

[B176] Sivan A. Corrales L. Hubert N. Williams J. B. Aquino-Michaels K. Earley Z. M. (2015). Commensal *Bifidobacterium* promotes antitumor immunity and facilitates anti-PD-L1 efficacy. *Science* 350 1084–1089. 10.1126/science.aac4255 26541606 PMC4873287

[B177] Sokol H. Pigneur B. Watterlot L. Lakhdari O. Bermudez-Humaran L. G. Gratadoux J. J. (2008). *Faecalibacterium prausnitzii* is an anti-inflammatory commensal bacterium identified by gut microbiota analysis of Crohn disease patients. *Proc. Natl. Acad. Sci. U.S.A.* 105 16731–16736. 10.1073/pnas.0804812105 18936492 PMC2575488

[B178] Sokol H. Seksik P. Furet J. P. Firmesse O. Nion-Larmurier I. Beaugerie L. (2009). Low counts of *Faecalibacterium prausnitzii* in colitis microbiota. *Inflamm. Bowel Dis.* 15 1183–1189. 10.1002/ibd.20903 19235886

[B179] St Jean A. T. Swofford C. A. Panteli J. T. Brentzel Z. J. Forbes N. S. (2014). Bacterial delivery of *Staphylococcus aureus* alpha-hemolysin causes regression and necrosis in murine tumors. *Mol. Ther.* 22 1266–1274. 10.1038/mt.2014.36 24590046 PMC4089002

[B180] Staley C. Weingarden A. R. Khoruts A. Sadowsky M. J. (2017). Interaction of gut microbiota with bile acid metabolism and its influence on disease states. *Appl. Microbiol. Biotechnol.* 101 47–64. 10.1007/s00253-016-8006-6 27888332 PMC5203956

[B181] Stidham R. W. Higgins P. D. R. (2018). Colorectal cancer in inflammatory bowel disease. *Clin. Colon Rectal. Surg.* 31 168–178. 10.1055/s-0037-1602237 29720903 PMC5929884

[B182] Strauss J. Kaplan G. G. Beck P. L. Rioux K. Panaccione R. Devinney R. (2011). Invasive potential of gut mucosa-derived *Fusobacterium nucleatum* positively correlates with IBD status of the host. *Inflamm. Bowel Dis.* 17 1971–1978. 10.1002/ibd.21606 21830275

[B183] Sun C. H. Li B. B. Wang B. Zhao J. Zhang X. Y. Li T. T. (2019). The role of *Fusobacterium nucleatum* in colorectal cancer: from carcinogenesis to clinical management. *Chronic Dis. Transl. Med.* 5 178–187. 10.1016/j.cdtm.2019.09.001 31891129 PMC6926109

[B184] Sun J. Kato I. (2016). Gut microbiota, inflammation and colorectal cancer. *Genes Dis.* 3 130–143. 10.1016/j.gendis.2016.03.004 28078319 PMC5221561

[B185] Swidsinski A. Dorffel Y. Loening-Baucke V. Theissig F. Ruckert J. C. Ismail M. (2011). Acute appendicitis is characterised by local invasion with *Fusobacterium nucleatum*/*necrophorum*. *Gut* 60 34–40. 10.1136/gut.2009.191320 19926616

[B186] Tamboli C. P. Neut C. Desreumaux P. Colombel J. F. (2004). Dysbiosis in inflammatory bowel disease. *Gut* 53 1–4. 10.1136/gut.53.1.1 14684564 PMC1773911

[B187] Tan C. R. Zhou L. El-Deiry W. S. (2016). Circulating tumor cells versus circulating tumor DNA in colorectal cancer: pros and cons. *Curr. Colorectal Cancer Rep.* 12 151–161. 10.1007/s11888-016-0320-y 27516729 PMC4976692

[B188] Tanoue T. Morita S. Plichta D. R. Skelly A. N. Suda W. Sugiura Y. (2019). A defined commensal consortium elicits CD8 T cells and anti-cancer immunity. *Nature* 565 600–605. 10.1038/s41586-019-0878-z 30675064

[B189] Taxman D. J. Swanson K. V. Broglie P. M. Wen H. Holley-Guthrie E. Huang M. T. (2012). Porphyromonas gingivalis mediates inflammasome repression in polymicrobial cultures through a novel mechanism involving reduced endocytosis. *J. Biol. Chem.* 287 32791–32799. 10.1074/jbc.M112.401737 22843689 PMC3463344

[B190] Tojo R. Suarez A. Clemente M. G. de los Reyes-Gavilan C. G. Margolles A. Gueimonde M. (2014). Intestinal microbiota in health and disease: role of bifidobacteria in gut homeostasis. *World J. Gastroenterol.* 20 15163–15176. 10.3748/wjg.v20.i41.15163 25386066 PMC4223251

[B191] Torre L. A. Bray F. Siegel R. L. Ferlay J. Lortet-Tieulent J. Jemal A. (2015). Global cancer statistics, 2012. *CA Cancer J. Clin.* 65 87–108. 10.3322/caac.21262 25651787

[B192] Turnbaugh P. J. Ley R. E. Hamady M. Fraser-Liggett C. M. Knight R. Gordon J. I. (2007). The human microbiome project. *Nature* 449 804–810. 10.1038/nature06244 17943116 PMC3709439

[B193] Turroni F. Duranti S. Milani C. Lugli G. A. van Sinderen D. Ventura M. (2019). *Bifidobacterium bifidum*: a key member of the early human gut microbiota. *Microorganisms* 7:544. 10.3390/microorganisms7110544 31717486 PMC6920858

[B194] Turroni F. Foroni E. O’Connell Motherway M. Bottacini F. Giubellini V. Zomer A. (2010). Characterization of the serpin-encoding gene of *Bifidobacterium breve* 210 B. *Appl. Environ. Microbiol.* 76 3206–3219. 10.1128/AEM.02938-09 20348296 PMC2869134

[B195] Turroni F. Milani C. Duranti S. Lugli G. A. Bernasconi S. Margolles A. (2020). The infant gut microbiome as a microbial organ influencing host well-being. *Ital. J. Pediatr.* 46:16. 10.1186/s13052-020-0781-0 32024556 PMC7003403

[B196] Turroni F. Peano C. Pass D. A. Foroni E. Severgnini M. Claesson M. J. (2012). Diversity of bifidobacteria within the infant gut microbiota. *PLoS ONE* 7:e36957. 10.1371/journal.pone.0036957 22606315 PMC3350489

[B197] Turroni F. Serafini F. Foroni E. Duranti S. O’Connell Motherway M. Taverniti V. (2013). Role of sortase-dependent pili of *Bifidobacterium bifidum* PRL2010 in modulating bacterium-host interactions. *Proc. Natl. Acad. Sci. U.S.A.* 110 11151–11156. 10.1073/pnas.1303897110 23776216 PMC3703987

[B198] Turroni F. Serafini F. Mangifesta M. Arioli S. Mora D. van Sinderen D. (2014). Expression of sortase-dependent pili of Bifidobacterium bifidum PRL2010 in response to environmental gut conditions. *FEMS Microbiol. Lett.* 357 23–33. 10.1111/1574-6968.12509 24947069

[B199] Uchugonova A. Zhang Y. Salz R. Liu F. Suetsugu A. Zhang L. (2015). Imaging the different mechanisms of prostate cancer cell-killing by tumor-targeting *Salmonella* typhimurium A1-R. *Anticancer Res.* 35 5225–5229. 26408681

[B200] Underhill D. M. Iliev I. D. (2014). The mycobiota: interactions between commensal fungi and the host immune system. *Nat. Rev. Immunol.* 14 405–416. 10.1038/nri3684 24854590 PMC4332855

[B201] Ursell L. K. Clemente J. C. Rideout J. R. Gevers D. Caporaso J. G. Knight R. (2012a). The interpersonal and intrapersonal diversity of human-associated microbiota in key body sites. *J. Allergy Clin. Immunol.* 129 1204–1208. 10.1016/j.jaci.2012.03.010 22541361 PMC3342686

[B202] Ursell L. K. Metcalf J. L. Parfrey L. W. Knight R. (2012b). Defining the human microbiome. *Nutr. Rev.* 70(Suppl. 1), S38–S44. 10.1111/j.1753-4887.2012.00493.x 22861806 PMC3426293

[B203] Vander M. G. Heiden Cantley L. C. Thompson C. B. (2009). Understanding the Warburg effect: the metabolic requirements of cell proliferation. *Science* 324 1029–1033. 10.1126/science.1160809 19460998 PMC2849637

[B204] Ventura M. Canchaya C. Tauch A. Chandra G. Fitzgerald G. F. Chater K. F. (2007). Genomics of *Actinobacteria*: tracing the evolutionary history of an ancient phylum. *Microbiol. Mol. Biol. Rev.* 71 495–548. 10.1128/MMBR.00005-07 17804669 PMC2168647

[B205] Verma R. Lee C. Jeun E. J. Yi J. Kim K. S. Ghosh A. (2018). Cell surface polysaccharides of Bifidobacterium bifidum induce the generation of Foxp3(+) regulatory T cells. *Sci. Immunol.* 3:eaat6975. 10.1126/sciimmunol.aat6975 30341145

[B206] Vetizou M. Pitt J. M. Daillere R. Lepage P. Waldschmitt N. Flament C. (2015). Anticancer immunotherapy by CTLA-4 blockade relies on the gut microbiota. *Science* 350 1079–1084. 10.1126/science.aad1329 26541610 PMC4721659

[B207] Villeger R. Lopes A. Carrier G. Veziant J. Billard E. Barnich N. (2019). Intestinal microbiota: a novel target to improve anti-tumor treatment? *Int. J. Mol. Sci.* 20:4584. 10.3390/ijms20184584 31533218 PMC6770123

[B208] Virchow R. (1989). Cellular pathology. As based upon physiological and pathological histology. Lecture XVI–Atheromatous affection of arteries. 1858. *Nutr. Rev.* 47 23–25. 10.1111/j.1753-4887.1989.tb02747.x 2649802

[B209] Waddington L. Cyr T. Hefford M. Hansen L. T. Kalmokoff M. (2010). Understanding the acid tolerance response of bifidobacteria. *J. Appl. Microbiol.* 108 1408–1420. 10.1111/j.1365-2672.2009.04540.x 19796122

[B210] Wang H. F. Li L. F. Guo S. H. Zeng Q. Y. Ning F. Liu W. L. (2016). Evaluation of antibody level against *Fusobacterium nucleatum* in the serological diagnosis of colorectal cancer. *Sci. Rep.* 6:33440. 10.1038/srep33440 27678333 PMC5039407

[B211] Warren R. L. Freeman D. J. Pleasance S. Watson P. Moore R. A. Cochrane K. (2013). Co-occurrence of anaerobic bacteria in colorectal carcinomas. *Microbiome* 1:16. 10.1186/2049-2618-1-16 24450771 PMC3971631

[B212] Weeks D. F. Katz D. S. Saxon P. Kubal W. S. (2010). Lemierre syndrome: report of five new cases and literature review. *Emerg. Radiol.* 17 323–328. 10.1007/s10140-010-0858-y 20135186

[B213] Weinberger M. Wu T. Rubin M. Gill V. J. Pizzo P. A. (1991). *Leptotrichia buccalis* bacteremia in patients with cancer: report of four cases and review. *Rev. Infect. Dis.* 13 201–206. 10.1093/clinids/13.2.201 2041949

[B214] Wollowski I. Rechkemmer G. Pool-Zobel B. L. (2001). Protective role of probiotics and prebiotics in colon cancer. *Am. J. Clin. Nutr.* 73(2 Suppl.), 451S–455S. 10.1093/ajcn/73.2.451s 11157356

[B215] Wong S. H. Zhao L. Zhang X. Nakatsu G. Han J. Xu W. (2017). Gavage of fecal samples from patients with colorectal cancer promotes intestinal carcinogenesis in germ-free and conventional mice. *Gastroenterology* 153 1621–1633.e6. 10.1053/j.gastro.2017.08.022 28823860

[B216] Wood L. M. Paterson Y. (2014). Attenuated *Listeria monocytogenes*: a powerful and versatile vector for the future of tumor immunotherapy. *Front. Cell Infect. Microbiol.* 4:51. 10.3389/fcimb.2014.00051 24860789 PMC4026700

[B217] Wrzosek L. Miquel S. Noordine M. L. Bouet S. Joncquel M. Chevalier-Curt J. (2013). *Bacteroides thetaiotaomicron* and Faecalibacterium prausnitzii influence the production of mucus glycans and the development of goblet cells in the colonic epithelium of a gnotobiotic model rodent. *BMC Biol.* 11:61. 10.1186/1741-7007-11-61 23692866 PMC3673873

[B218] Wu T. Cen L. Kaplan C. Zhou X. Lux R. Shi W. (2015). Cellular components mediating coadherence of *Candida albicans* and *Fusobacterium nucleatum*. *J. Dent. Res.* 94 1432–1438. 10.1177/0022034515593706 26152186 PMC4577983

[B219] Yachida S. Mizutani S. Shiroma H. Shiba S. Nakajima T. Sakamoto T. (2019). Metagenomic and metabolomic analyses reveal distinct stage-specific phenotypes of the gut microbiota in colorectal cancer. *Nat. Med.* 25 968–976. 10.1038/s41591-019-0458-7 31171880

[B220] Yan F. Polk D. B. (2011). Probiotics and immune health. *Curr. Opin. Gastroenterol.* 27 496–501. 10.1097/MOG.0b013e32834baa4d 21897224 PMC4006993

[B221] Yang Y. (2015). Cancer immunotherapy: harnessing the immune system to battle cancer. *J. Clin. Invest.* 125 3335–3337. 10.1172/JCI83871 26325031 PMC4588312

[B222] Yatsunenko T. Rey F. E. Manary M. J. Trehan I. Dominguez-Bello M. G. Contreras M. (2012). Human gut microbiome viewed across age and geography. *Nature* 486 222–227. 10.1038/nature11053 22699611 PMC3376388

[B223] Yoshimoto S. Loo T. M. Atarashi K. Kanda H. Sato S. Oyadomari S. (2013). Obesity-induced gut microbial metabolite promotes liver cancer through senescence secretome. *Nature* 499 97–101. 10.1038/nature12347 23803760

[B224] Yu L. C. (2018). Microbiota dysbiosis and barrier dysfunction in inflammatory bowel disease and colorectal cancers: exploring a common ground hypothesis. *J. Biomed. Sci.* 25:79. 10.1186/s12929-018-0483-8 30413188 PMC6234774

[B225] Yu R. Zuo F. Ma H. Chen S. (2019). Exopolysaccharide-producing bifidobacterium adolescentis strains with similar adhesion property induce differential regulation of inflammatory immune response in Treg/Th17 axis of DSS-colitis mice. *Nutrients* 11:782. 10.3390/nu11040782 30987344 PMC6520857

[B226] Yu T. Guo F. Yu Y. Sun T. Ma D. Han J. (2017). *Fusobacterium nucleatum* promotes chemoresistance to colorectal cancer by modulating autophagy. *Cell* 170 548–563.e16. 10.1016/j.cell.2017.07.008 28753429 PMC5767127

[B227] Yu W. D. Sun G. Li J. Xu J. Wang X. (2019). Mechanisms and therapeutic potentials of cancer immunotherapy in combination with radiotherapy and/or chemotherapy. *Cancer Lett.* 452 66–70. 10.1016/j.canlet.2019.02.048 30902563

[B228] Zhang K. Dai H. Liang W. Zhang L. Deng Z. (2019). Fermented dairy foods intake and risk of cancer. *Int. J. Cancer* 144 2099–2108. 10.1002/ijc.31959 30374967

[B229] Zhang M. Swofford C. A. Forbes N. S. (2014). Lipid A controls the robustness of intratumoral accumulation of attenuated *Salmonella* in mice. *Int. J. Cancer* 135 647–657. 10.1002/ijc.28700 24374783

[B230] Zitvogel L. Ma Y. Raoult D. Kroemer G. Gajewski T. F. (2018). The microbiome in cancer immunotherapy: diagnostic tools and therapeutic strategies. *Science* 359 1366–1370. 10.1126/science.aar6918 29567708

[B231] Zou W. Chen L. (2008). Inhibitory B7-family molecules in the tumour microenvironment. *Nat. Rev. Immunol.* 8 467–477. 10.1038/nri2326 18500231

